# An optimization model for fleet sizing and empty pallet allocation considering CO_2_ emissions

**DOI:** 10.1371/journal.pone.0229544

**Published:** 2020-02-21

**Authors:** Jianwei Ren, Chunhua Chen, Jian Gao, Chenxi Feng

**Affiliations:** 1 Transportation Institute, Inner Mongolia University, Hohhot, Inner Mongolia, China; 2 School of Mathematical Sciences, Inner Mongolia University, Hohhot, Inner Mongolia, China; 3 Faculty of Business, University of Plymouth, Plymouth, Devon, England, United Kingdom; 4 School of Business Administration, Jiangxi University of Finance and Economics, Nanchang, Jiangxi, China; 5 Inner Mongolia Branch of Agricultural Bank of China, Hohhot, Inner Mongolia, China; Univerza v Mariboru, SLOVENIA

## Abstract

Pallets are the most common equipment for transporting and storing goods. More and more companies are willing to rent pallets. Pallet rental companies need to transport pallets from their pallet rental service stations to customers and take these pallets back when they are unloaded. Hence, managers should scientifically configure vehicles for their pallet rental service stations. The fleet size, which indicates the amount and types of vehicles, can significantly affect the efficiency and costs of empty pallet allocation. Therefore, an optimization model for fleet sizing and empty pallet allocation is proposed using the methods of mixed-integer programming and stochastic programming. The objectives of this model are to maximize the profits of pallet rental companies and minimize carbon dioxide (CO_2_) emissions from vehicles. A particle swarm optimization algorithm with inertia weight (IPSO) is developed to solve the proposed model because IPSO can avoid becoming trapped in local optima and is able to find a globally optimal solution within a reasonable number of iterations. A numerical example proves the effectiveness of the proposed model and IPSO. The results of numerical tests show that the amount of CO_2_ emissions from vehicles can affect the decision on fleet sizing and empty pallet allocation. However, if the price, rental fees, or idle costs of the vehicles with low CO_2_ emissions are too high, managers would not choose them.

## Introduction

Pallets are the most common equipment for transporting and storing goods. They are widely used not only in developed countries but also in developing countries. About 2 billion pallets are used in the United States, and 4 billion pallets are in circulation in Europe [1]. In China, there are more than 1.26 billion pallets according to the China Federation of Logistics Standardization.

Purchasing pallets or renting pallets? Although owning pallets can make a company specify the exact pallets' size, quality, and material for loading their products, more and more companies are willing to rent pallets [2]. The advantages of renting pallets are as follows. (1) Users can save a large upfront cost for pallets. (2) Users do not have to keep idle pallets in a warehouse to use during those few weeks of seasonal busy periods. (3) Users can be free to develop their core business without the distraction of pallet management. There exist many pallet rental companies in the world. In the United States, there are CHEP (Commonwealth Handling Equipment Pool), iGPS (Intelligent Global Pooling Systems), and PECO (PECO pallet pooling Co. Ltd.) pallet rental systems. IPP Logipal and LPR (La Pallet Rouge) are the two major pallet rental service providers in Europe. JPR (Japan Pallet Rental Corporation) and KPR (Korea Pallet Pool Co., Ltd.) provide pallet rental services in Japan and South Korea, respectively. An interesting thing is that rental pallets are usually painted to identify them from non-rental pallets. The pallets of CHEP, PECO, and iGPS are blue, red, and black, respectively.

Because the pallet rental industry plays an important role in supply chain management, researchers have studied it from several perspectives. Ray et al. [3], Roy et al. [4], Lacefield [5], Mosqueda [6], and Ren et al. [7] analyzed the costs of renting and purchasing pallets. Because the results are different, we advise that managers should decide whether to rent or purchase pallets based on their actual situation. Ren et al. [8] applied a DEA approach to evaluate the performance of pallet rental companies. Pallet rental companies have been trying to use RFID (Radio Frequency Identification) technology to track pallets because the loss rate of pallets is very high [9–12]. Gnoni and Rollo [13], Kim and Glock [14], and Ren et al. [15] researched how to develop an advanced tracking system using RFID. Elia and Gnoni [16], Xu [17], and Li et al. [18] also made the effort to understand how to efficiently manage pallets taking advantage of information technology. Ren et al. [19], Doungpattra et al. [20], Zhou et al. [21], and Kesen and Alim [22] developed some optimization models for empty pallet allocation. Bilbao et al. [23], Carrano et al. [24], Tornese et al. [25], Bengtssona and Logie [26], Tornese et al. [27], Accorsi et al. [28], and Kočí [29] measured carbon emissions from pallet logistics and proposed that pallet rental system was the most environmentally friendly pallet management mode.

To the best of our knowledge, this is the first study to address the joint optimization of fleet sizing and empty pallet allocation. The contributions of this paper are as follows. (1) Using the methods of mixed-integer programming and stochastic programming, we develop a mathematical model for the joint optimization of fleet sizing and empty pallet allocation considering carbon dioxide (CO_2_) emissions. (2) A particle swarm optimization algorithm with inertia weight (IPSO) is developed to solve the proposed model.

The paper is organized as follows. In section 2, the problem is analyzed. In section 3, the optimization model and solution method for the problem are proposed. A numerical example is introduced in section 4. Conclusions are drawn in section 5.

## Problem description

**Fig 1:**
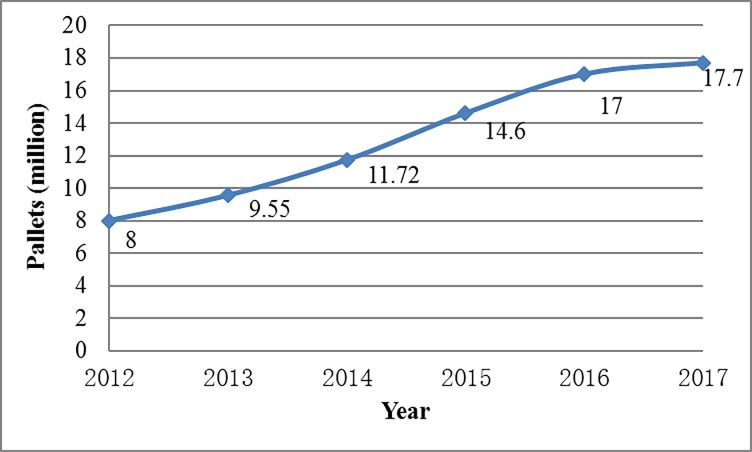
Total rental pallets in China from 2012 to 2017.

The proportion of total pallets occupied by rental pallets is increasing year by year. The number of rental pallets is more than 300 million in the United States. In China, as shown in Fig 1, the number of rental pallets has been increasing rapidly. In 2017, they accounted for about 1.4% of China’s total pallets [30].

Pallet rental companies need to transport pallets from their pallet rental service stations (PRSSs) to customers and take these pallets back when they are unloaded, so managers should configure the right number of vehicles for PRSSs. Too many vehicles would increase the burden of pallet rental companies, while too few vehicles may make it difficult to meet customers' demands on time. Moreover, what types of vehicles should be selected? It is also a troublesome matter because the carrying capacity of each kind of vehicle is different.

**Fig 2:**
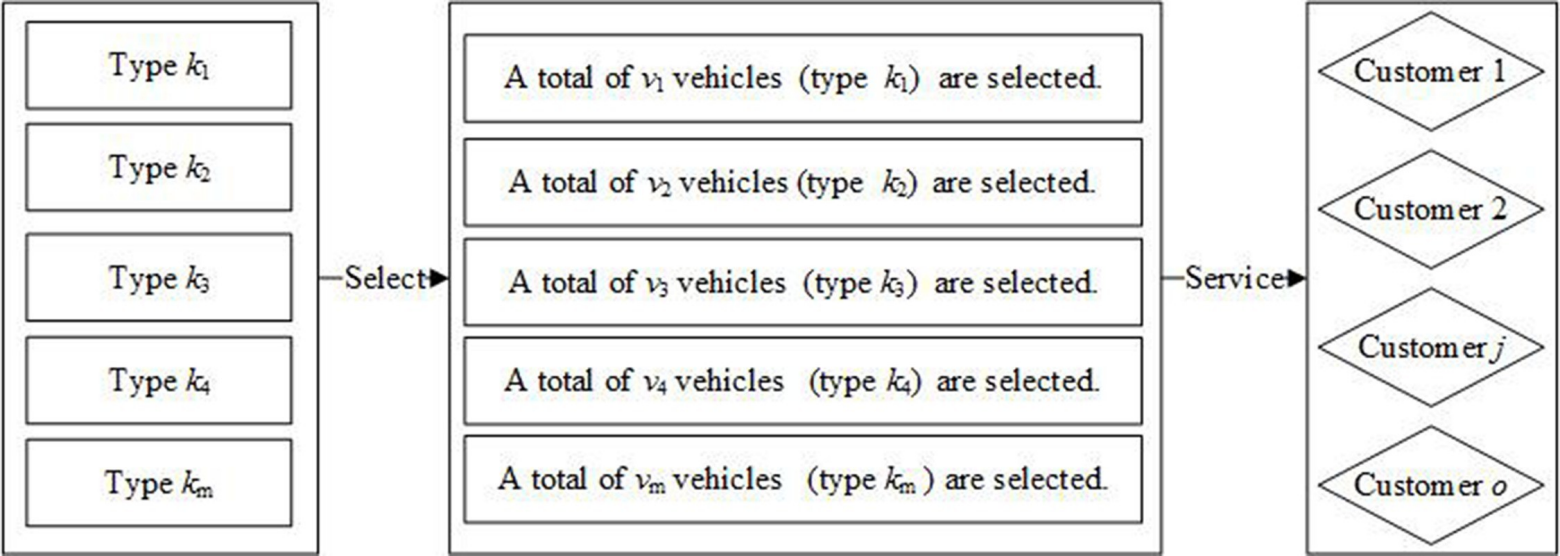
The problem of fleet sizing.

**Fig 3:**
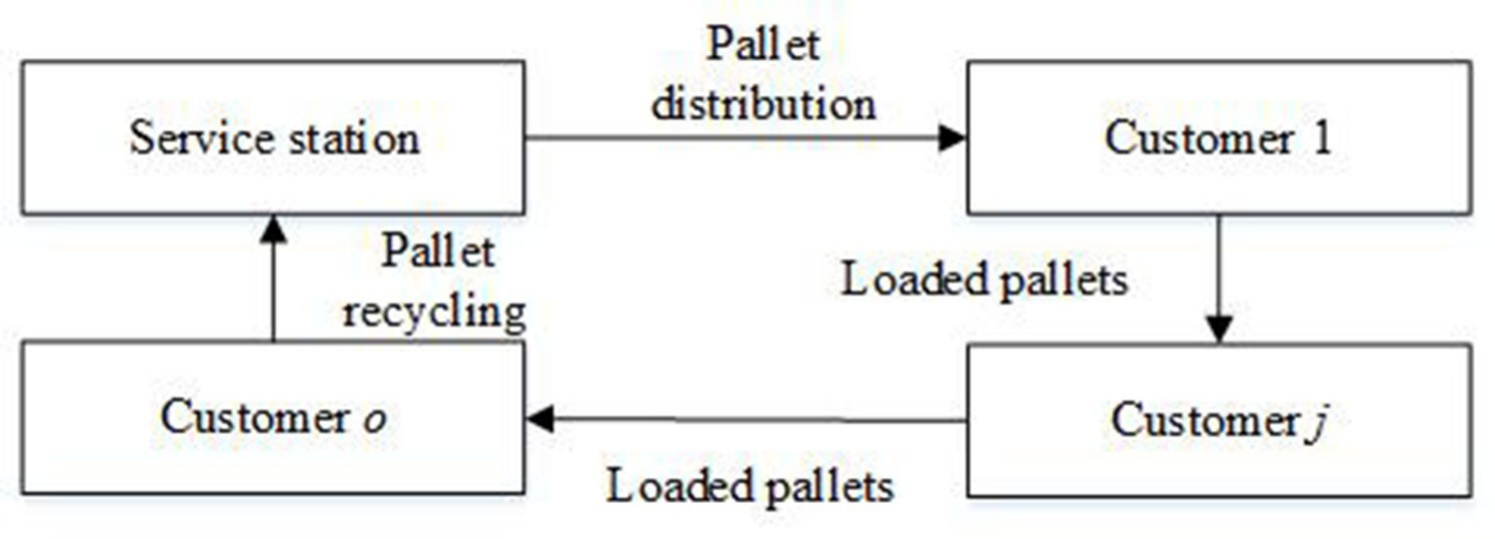
Pallets are used across a supply chain.

The problem of fleet sizing means that pallet rental companies’ managers decide on the amount (*v*) and types (*k*) of vehicles at PRSSs, which is shown in Fig 2. The fleet size can significantly affect the efficiency and costs of empty pallet allocation that refers to empty pallet distribution and empty pallet recycling. Pallet rental companies should use vehicles to transport empty pallets from PRSSs to customers when these customers require pallets (distribution). Then, these pallets are loaded goods and moved to these customers' customers. Finally, these pallets should be taken back from the last customer by vehicles (recycling). These processes are shown in Fig 3. Note that pallet rental companies do not need to manage the loaded pallets.

## Methodology

**Fig 4:**
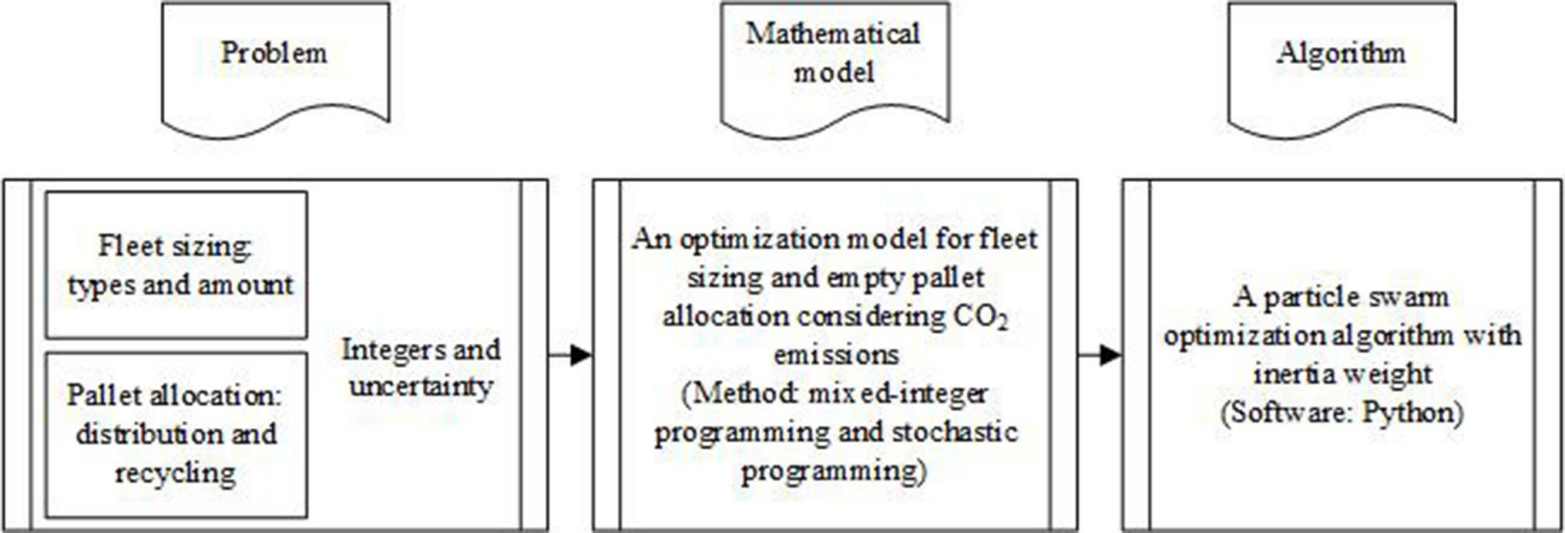
The conceptual framework.

Both the number of pallets and the number of vehicles can only take non-negative integer values, and decision-makers have to take uncertain demands into account when they make decisions. Therefore, the optimization model for fleet sizing and empty pallet allocation is developed using the methods of mixed-integer programming and stochastic programming. The joint optimization of fleet sizing and empty pallet allocation is NP-hard (nondeterministic polynomial). Thus, we develop a particle swarm optimization algorithm with inertia weight (IPSO) to solve the proposed model. The conceptual framework of this research is shown in Fig 4.

### 3.1 Mathematical model

We let *t*∈*T*, *p*∈*P*, k∈*K*, *i*∈*I*, *j*∈*J*, and *o*∈*O* denote the time period, the pallet type, the vehicle type, the pallet rental service station, the demand area in which customers require pallets, and the supply area in which customers need pallet rental companies to retrieve pallets, respectively. There are six sets of decision variables. *v*_*ik*_ is the number of *k* vehicles configured for *i*. $X_{ijp}^{t}$ ($X_{oip}^{t}$) indicates the number of *p* pallets moved from *i* to *j* (from *o* to *i*) in period *t*, and $v_{ijk}^{t}$ ($v_{oik}^{t}$) represents the number of *k* vehicles used to transport these pallets. If there are not enough vehicles to transport pallets, managers can rent vehicles from rental companies. Thus, we let $\theta_{ik}^{t}$ denote the number of *k* vehicles rented by *i* in period *t*.

The objective function (1) maximizes the profits of pallet rental companies because managers always pay much attention to profits when they make decisions. Profit is the amount of income ($\sum_{i\in I}\sum_{j\in J}\sum_{p\in P}\sum_{t\in T}\;\xi_{p}X_{ijp}^{t}$) that remains after accounting for all costs (the rest of the right side of the formula (1)), i.e., the costs of purchasing vehicles, pallet distribution costs, pallet recycling costs, pallet storage costs, pallet loading and unloading costs, idle costs of vehicles, and rental fees of vehicles. Idle costs of vehicles are considered because pallet rental companies have to pay the maintenance fee, capital cost, and some other costs even if the purchased vehicles are not in use. In Eq (1), *ξ*_*p*_ indicates the rental fee of a *p* pallet; *C*_*k*_ represents the price of a *k* vehicle; E_*k*_ denotes the transportation cost of a *k* vehicle per kilometer; $g_{ijk}^{t}$ ($g_{oik}^{t}$) represents how many times a *k* vehicle can run from *i* to *j* (from *o* to *i*) in period *t*, and *l*_*ij*_ (*l*_*oi*_) indicates the related distance; $M_{ip}^{t}$ is the storage of *p* pallets at *i* in period *t*, and *H*_*ip*_ is the related unitary cost; Λ_*p*_ represents the loading and unloading cost of a *p* pallet; *β*_k_ and *α*_k_ are the idle cost and rental fee of a *k* vehicle, respectively.

$$\begin{array}{l} \max R=\sum_{i\in I}\sum_{j\in J}\sum_{p\in P}\sum_{t\in T}\;\xi_{p}X_{ijp}^{t}-\sum_{i\in I}\sum_{k\in K}C_{k}v_{ik}-\sum_{i\in I}\sum_{j\in J}\sum_{k\in K}\sum_{t\in T}\;E_{k}g_{ijk}^{t}l_{ij}v_{ijk}^{t}-\\ \;\sum_{i\in I}\sum_{o\in O}\sum_{k\in K}\sum_{t\in T}\;E_{k}g_{oik}^{t}l_{oi}v_{oik}^{t}\;-\sum_{i\in I}\sum_{p\in P}\sum_{t\in T}\;H_{ip}\;M_{ip}^{t}-\sum_{i\in I}\sum_{j\in J}\sum_{p\in P}\sum_{t\in T}\;\Lambda_{p}X_{ijp}^{t}-\\ \;\sum_{i\in I}\sum_{o\in O}\sum_{p\in P}\sum_{t\in T}\;\Lambda_{p}X_{oip}^{t}-\sum_{i\in I}\sum_{k\in K}\sum_{t\in T}\;\beta_{k}\max\left\{v_{ik}+\theta_{ik}^{t}-\sum_{j\in J}v_{ijk}^{t},0\right\}-\sum_{i\in I}\sum_{k\in K}\sum_{t\in T}\;\alpha_{k}\theta_{ik}^{t} \end{array}$$ (1)

The objective function (2) minimizes the CO_2_ emissions from vehicles. Managers have to understand the environmental impacts of pallet logistics because there are many pallets. According to the United States Environmental Protection Agency, transportation occupied 29% (the largest portion) of total United States greenhouse gas (GHG) emissions in 2017, and CO_2_ accounted for 97% (the largest portion) of total transportation sector GHG [31]. Each *k* vehicle emits *B*_*k*_ CO_2_ per kilometer and travels $l_{ij}g_{ijk}^{t}$ (or $l_{oi}g_{oik}^{t}$) kilometers, so the minimum total CO_2_ emissions from all vehicles are

$$\min B=\sum_{i\in I}\sum_{j\in J}\sum_{k\in K}\sum_{t\in T}\;B_{k}l_{ij}g_{ijk}^{t}v_{ijk}^{t}+\sum_{i\in I}\sum_{o\in O}\sum_{k\in K}\sum_{t\in T}\;B_{k}l_{oi}g_{oik}^{t}\;v_{oik}^{t}$$ (2)

The constraint set (3) ensures that pallet rental companies cannot move extra pallets to customers. In other words, they can only meet customers’ deterministic ($D_{jp}^{t}$) and uncertain ($\Gamma_{jp}^{t}$) demands.

$$\sum_{i\in I}\;X_{ijp}^{t}\leq D_{jp}^{t}+\Gamma_{jp}^{t}$$ (3)

The constraint set (4) guarantees that there are enough vehicles to transport pallets between PRSSs and demand areas. In other words, the total number of transported pallets cannot exceed the carrying capacity of all vehicles that has been assigned. Each *k* vehicle has a carrying capacity of *z*_*k*_ and can run $g_{ijk}^{t}$ times, so the total carrying capacity is $\sum_{k\in K}\;g_{ijk}^{t}z_{k}v_{ijk}^{t}$. *ψ*_*p*_ represents the carrying capacity occupied by a *p* pallet.

$$\sum_{k\in K}\;g_{ijk}^{t}z_{k}v_{ijk}^{t}\geq \sum_{p\in P}\;\psi_{p}X_{ijp}^{t}$$ (4)

According to the constraint set (5), pallet rental companies should retrieve all pallets in supply areas. $S_{op}^{t}$ and ${ϒ}_{op}^{t}$ denote the certain and uncertain pallets that should be taken back, respectively.

$$\sum_{i\in I}X_{oip}^{t}=S_{op}^{t}+{ϒ}_{op}^{t}$$ (5)

The constraint set (6) ensures that there are enough vehicles to transport pallets between supply areas and PRSSs, which is similar to the constraint set (4).

$$\sum_{k\in K}\;g_{oik}^{t}z_{k}v_{oik}^{t}\geq \sum_{p\in P}\;\psi_{p}X_{oip}^{t}$$ (6)

According to the constraint set (7), the number of pallets that are transported from a PRSS to all demand areas cannot exceed the PRSS’s supply capacity, i.e., the sum of the pallet storage in the last period ($M_{ip}^{t-1}$) and the purchased pallets in this period ($S_{ip}^{t}$).

$$\sum_{j\in J}X_{ijp}^{t}\leq M_{ip}^{t-1}+S_{ip}^{t}$$ (7)

According to the constraint set (8), the storage of pallets at a PRSS ($M_{ip}^{t}$) is from the storage in the last period ($M_{ip}^{t-1}$), the purchased pallets ($S_{ip}^{t}$), the pallets that are taken back from all supply areas ($\sum_{o\in O}X_{oip}^{t}$), and the negative number of pallets that are moved from this PRSS to all demand areas ($-\sum_{j\in J}X_{ijp}^{t}$).

$$M_{ip}^{t}=M_{ip}^{t-1}+S_{ip}^{t}+\sum_{o\in O}X_{oip}^{t}-\sum_{j\in J}X_{ijp}^{t}$$ (8)

The constraint set (9) imposes an upper storage capacity (Ψ_*i*_) on the number of the pallets that can be stored at a PRSS, where *υ*_*p*_ indicates the storage capacity occupied by a *p* pallet.

$$\sum_{p\in P}\upsilon_{p}M_{ip}^{t}\leq \Psi_{i}$$ (9)

According to the constraint sets (10) and (11), the number of vehicles that are used to distribute or recycle pallets cannot be more than the available vehicles, i.e., the sum of the vehicles that are configured at this PRSS (*v*_*ik*_) and the rented vehicles ($\theta_{ik}^{t}$). We assume that managers can efficiently use vehicles. For example, managers can retrieve pallets using vehicles’ return trips.

$$\sum_{j\in J}\;v_{ijk}^{t}\leq v_{ik}+\theta_{ik}^{t}$$ (10)

$$\sum_{o\in O}\;v_{oik}^{t}\leq v_{ik}+\theta_{ik}^{t}$$ (11)

The constraint set (12) guarantees that all decision variables can only take non-negative integer values.

$$X_{ijp}^{t},X_{oip}^{t},v_{ijk}^{t},v_{oik}^{t},v_{ik},\;\theta_{ik}^{t}\geq 0,and\;\mathrm{int}$$ (12)

### 3.2 Solution method

In order to solve the model, we should transform the multiple objective functions into a single objective function. First, we need to propose the objective function (13) instead of the objective function (2), which means that the problem of minimizing CO_2_ emissions is turned into a problem of minimizing the costs of CO_2_ emissions. *ρ* is the unitary cost of CO_2_ emissions.

$$\min B^{'}=\sum_{i\in I}\sum_{j\in J}\sum_{k\in K}\sum_{t\in T}\;\rho B_{k}g_{ijk}^{t}l_{ij}v_{ijk}^{t}+\sum_{i\in I}\sum_{o\in O}\sum_{k\in K}\sum_{t\in T}\;\rho B_{k}g_{oik}^{t}l_{oi}v_{oik}^{t}$$ (13)

Then, we can use the objective function (14) instead of the objective function (13), which indicates that the minimization problem is turned into a maximization problem.

$$\max B^{''}=-(\sum_{i\in I}\sum_{j\in J}\sum_{k\in K}\sum_{t\in T}\;\rho B_{k}g_{ijk}^{t}l_{ij}v_{ijk}^{t}+\sum_{i\in I}\sum_{o\in O}\sum_{k\in K}\sum_{t\in T}\;\rho B_{k}g_{oik}^{t}l_{oi}v_{oik}^{t})$$ (14)

Finally, the new objective function of the proposed model is represented by formula (15).

$$\begin{array}{l} \max R^{'}=\sum_{i\in I}\sum_{j\in J}\sum_{p\in P}\sum_{t\in T}\;\xi_{p}X_{ijp}^{t}-\sum_{i\in I}\sum_{k\in K}\;C_{k}v_{ik}-\sum_{i\in I}\sum_{j\in J}\sum_{k\in K}\sum_{t\in T}\;E_{k}g_{ijk}^{t}l_{ij}v_{ijk}^{t}-\\ \;\sum_{i\in I}\sum_{o\in O}\sum_{k\in K}\sum_{t\in T}\;E_{k}g_{oik}^{t}l_{oi}v_{0ik}^{t}-\sum_{i\in I}\sum_{p\in P}\sum_{t\in T}\;H_{ip}\;M_{ip}^{t}-\sum_{i\in I}\sum_{j\in J}\sum_{p\in P}\sum_{t\in T}\;\Lambda_{p}X_{ijp}^{t}-\\ \;\sum_{i\in I}\sum_{o\in O}\sum_{p\in P}\sum_{t\in T}\;\Lambda_{p}X_{oip}^{t}-\sum_{i\in I}\sum_{k\in K}\sum_{t\in T}\;\beta_{k}\max\left\{v_{ik}+\theta_{ik}^{t}-\sum_{j\in J}v_{ijk}^{t},0\right\}-\\ \;\sum_{i\in I}\sum_{k\in K}\sum_{t\in T}\;\alpha_{k}\theta_{ik}^{t}-\sum_{i\in I}\sum_{j\in J}\sum_{k\in K}\sum_{t\in T}\;\rho B_{k}g_{ijk}^{t}l_{ij}v_{ijk}^{t}-\sum_{i\in I}\sum_{o\in O}\sum_{k\in K}\sum_{t\in T}\;\rho B_{k}g_{oik}^{t}l_{oi}v_{oik}^{t} \end{array}$$ (15)

**Fig 5:**
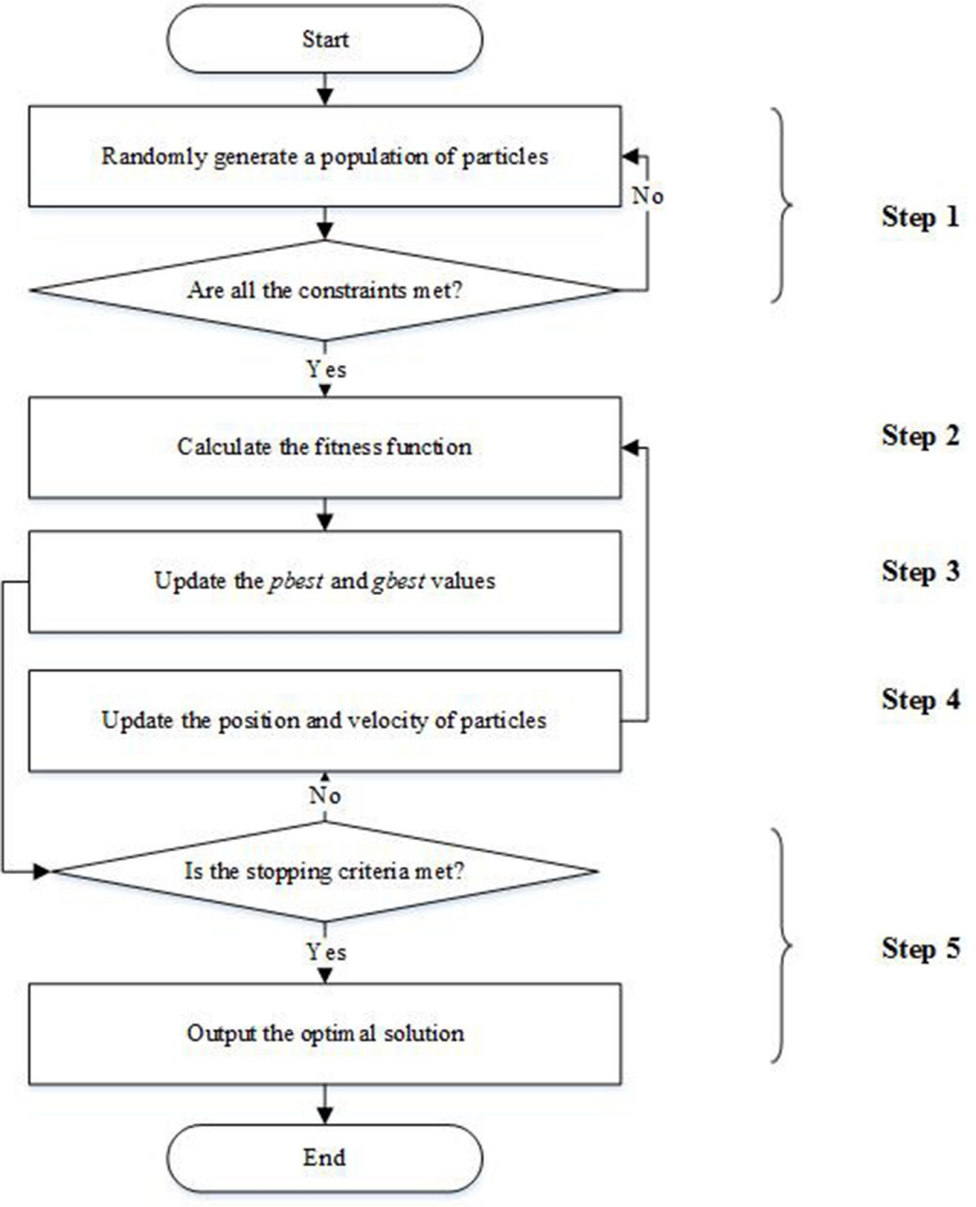
The flowchart of IPSO.

The particle swarm optimization (PSO) is a well-known stochastic optimization algorithm in which particles evolve to find the best solution by cooperation and competition among each other. Compared with other heuristic algorithms, e.g., genetic algorithm and ant colony algorithm, PSO has many significant advantages as follows. (1) PSO has few parameters needed to be set and is simple and easy to understand. (2) PSO can quickly converge to a satisfactory solution by the random search strategy [32]. Therefore, PSO has been applied in many fields including logistics, finance, and engineering [33]. However, classical PSO is likely to fall into local optima. Hence, some modified PSO algorithms have been proposed. A particle swarm optimization algorithm with inertia weight (IPSO) is used to solve the optimization model for fleet sizing and empty pallet allocation [34]. IPSO can avoid falling into local optima because it can control the velocity by setting inertia factor. Moreover, IPSO is able to find a globally optimal solution within a reasonable number of iterations. Therefore, IPSO is an effective and efficient algorithm for solving our NP-hard problem. The flowchart of IPSO is shown in Fig 5.

Step 1. Randomly generate a population of particles. The information of each particle *i* (*i*∈*I*)can be represented by *J* dimensional vectors. The initial position of particle *i* is $p_{ij}^{o}=\left|\mathrm{int}(N\times rnd_{ij}^{p})\right|$ (*j*∈*J*), where *N* depends on the constraints of the problem and $rnd_{ij}^{p}$ is a random value in [0, 1]. The initial velocity of particle *i* is $v_{ij}^{o}=M\times rnd_{ij}^{v}-\frac{M}{2}$, where *M* is assigned the value of 10 and $rnd_{ij}^{v}$ is a random value in [0, 1]. If the constraint sets (3)-(12) are met, go to the next step. Otherwise, generate another population of particles.
Step 2. Calculate the fitness function *f*(*i*) = *f*_1_(*i*)+*f*_2_(*i*). *f*_1_(*i*) is the objective function of the proposed model (formula 15), and *f*_2_(*i*) is calculated by $f_{2}(i)=\sum_{k\in K}\sum_{i\in I}\sum_{j\in J}P_{k}\times s_{k}$. $P_{k}=\left\{ \begin{array}{l} U_{k},\;\mathrm{if}\;k-th\;\mathrm{constraint}\;\mathrm{is}\;\mathrm{not}\;\mathrm{met}\\ 0,\;otherwise \end{array}\right.$, where *U*_*k*_ indicates the penalty coefficient. We set *U*_*k*_ = −700000 for constraint sets (4) and (6) while *U*_*k*_ = −120 for the other constraints because we hope all constraints are met, especially the constraint sets (4) and (6). *s*_*k*_ = |b_*k*_−*g*_*k*_(*p*_*ij*_)|, where *g*_*k*_(*p*_*ij*_)≤(or = ,≥) b_*k*_ represents the *k*−*th* constraint.
Step 3. Update the *pbest* and *gbest* by $\left\{ \begin{array}{l} if\;f(i)>f(pbest_{ij})\;then\;pbest_{ij}=p_{ij}\\ if\;f(i)>f(gbest_{j})\;then\;gbest_{j}=p_{ij} \end{array}\right.$, where *pbest*_*ij*_ indicates the best position of particle *i* while *gbest*_*j*_ is the best position of the whole particles.
Step 4. Position and velocity are updated with $p_{ij}^{n}=p_{ij}^{o}+v_{ij}^{n}$ and $v_{ij}^{n}=w\times v_{ij}^{o}+c1\times rnd_{1}\times (pbest_{ij}-p_{ij}^{o})+c2\times rnd_{2}\times (gbest_{j}-p_{ij}^{o})$, respectively. $w=w_{\min}+(w_{\max}-w_{\min})\frac{n_{\max}‑n}{n_{\max}}$ is the inertia factor, where *n* is the iteration index; *n*_max_ is the maximum number of iterations; *w*_min_ = 0.4; and *w*_max_ = 0.9. The inertia factor plays a significant role in balancing the global search and local search [34]. When *w* is small, IPSO converges quickly but may fall into local optima. When *w* is large, IPSO may find globally optimal solutions but convergences slowly. Our approach can find global optima within an acceptable number of iterations because *w* is large at the beginning of the iteration and small in the later iterations. $c1=(c_{1s}\;‑c_{1d})\times \frac{n_{\max}-n}{n_{\max}}+c_{1d}$ is self-confidence, while $c2=(c_{2s}\;‑c_{2d})\times \frac{n_{\max}-n}{n_{\max}}+c_{2d}$ indicates swarm confidence. c_1*s*_ = 2.5, c_1*d*_ = 0.5, c_2*s*_ = 0.5, c_2*d*_ = 2.5 [35]. Both *rnd*_1_ and *rnd*_2_ are random values in [0, 1]. We set *v*_max_ = 10 and *v*_min_ = −10. If $v_{ij}^{n}>v_{\max}$, then $v_{ij}^{n}=v_{\max}$. If $v_{ij}^{n}<v_{\min}$, then $v_{ij}^{n}=v_{\min}$.
Step 5. Stop the IPSO when the stopping criteria is satisfied (*n*_max_ = 2000). Otherwise, go to step 4.

## Numerical example and analysis

### 4.1 Numerical example

A company is planning to provide pallet rental services, and its managers have to decide how to configure vehicles for its service stations including *i*^1^, *i*^2^, and *i*^3^. The information about the three stations and alternative vehicles is shown in Tables 1 and 2. The length of time periods is 5 (*t*^1^,*t*^2^,…,*t*^5^). This company provides two kinds of pallets, i.e., *p*^1^ and *p*^2^. Their carrying capacity (and inventory capacity) occupancy factors are 1 and 1.1, unitary rental fees are 72 CNY per period and 108 CNY per period, and unitary loading and unloading costs are 0.12 CNY and 0.14 CNY, respectively. This company has signed service agreements with some customers and the market department has estimated uncertain demands, as shown in Table 3. The unitary cost of CO_2_ emissions is 0.00004186 CNY/g (the average price in January 2019 in China). The other parameters are shown in Table 4.

**Table 1:** Parameters of PRSSs.

Station	The purchased *p*^1^ pallets (*t*^1^/*t*^2−3^/*t*^4−5^)	The purchased *p*^2^ pallets (*t*^1^/*t*^2−3^/*t*^4−5^)	Storage capacity	Storage cost (*p*^1^/*p*^2^, CNY)
*i*^1^	4000/3/23	4000/4/40	60000	0.1/0.12
*i*^2^	4000/3/23	4000/4/40	80000	0.2/0.4
*i*^3^	4000/3/23	4000/4/40	80000	0.2/0.4

**Table 2:** Parameters of alternative vehicles.

Type	Carrying capacity	CO_2_ emissions (g/km)	Transportation cost (CNY/km)	Idle cost (CNY)	Rental fee (CNY)	Price (CNY)
*k*^1^	400	598.03	0.75	100	80000	400000
*k*^2^	300	514.03	0.6	90	60000	300000
*k*^3^	200	501.64	0.55	80	50000	200000
*k*^4^	120	326.88	0.4	70	30000	90000
*k*^5^	120	175	0.4	70	30000	90000

**Table 3:** Demands.

Area	Deterministic demands of *p*^1^ pallets (*t*^1^/*t*^2−3^/*t*^4−5^)	Deterministic demands of *p*^2^ pallets (*t*^1^/*t*^2−3^/*t*^4−5^)	Uncertain demands of *p*^1^ pallets (*t*^1^/*t*^2−3^/*t*^4−5^)	Uncertain demands of *p*^2^ pallets (*t*^1^/*t*^2−3^/*t*^4−5^)
*j*^1^	2000/2200/2000	2000/3000/3000	~/~/*N*(200,4) *	~/~/ *N*(400,16)
*j*^2^	1600/1600/1500	1500/2000/2000	~/~/~	~/~/~
*j*^3^	1500/1400/1500	1800/1800/1600	~/~/~	~/~/~
*j*^4^	1200/1300/1300	1800/1800/1800	~/~/~	~/~/~
*o*^1^	-6300/-6500/-6300	-7100/-8600/-8400	~/~/−*N*(200,4)	~/~/−*N*(400,16)

* N(100,4) *means that the demand is distributed normally with mean 100 and variance 4*. *Negative number indicates that customers have pallets needed to be taken away*. *~ represents that customers do not need pallets or have no pallets needed to be taken away*.

**Table 4:** Distance and turnover times between PRSSs and demand (or supply) areas.

Area	*i*^1^	*i*^2^	*i*^3^
	Distance(km)/ turnover times		
*j*^1^	50/10	55/10	20/15
*j*^2^	60/10	55/10	30/15
*j*^3^	40/15	40/15	30/15
*j*^4^	65/10	60/10	40/15
*o*^1^	70/10	75/10	50/10

**Fig 6:**
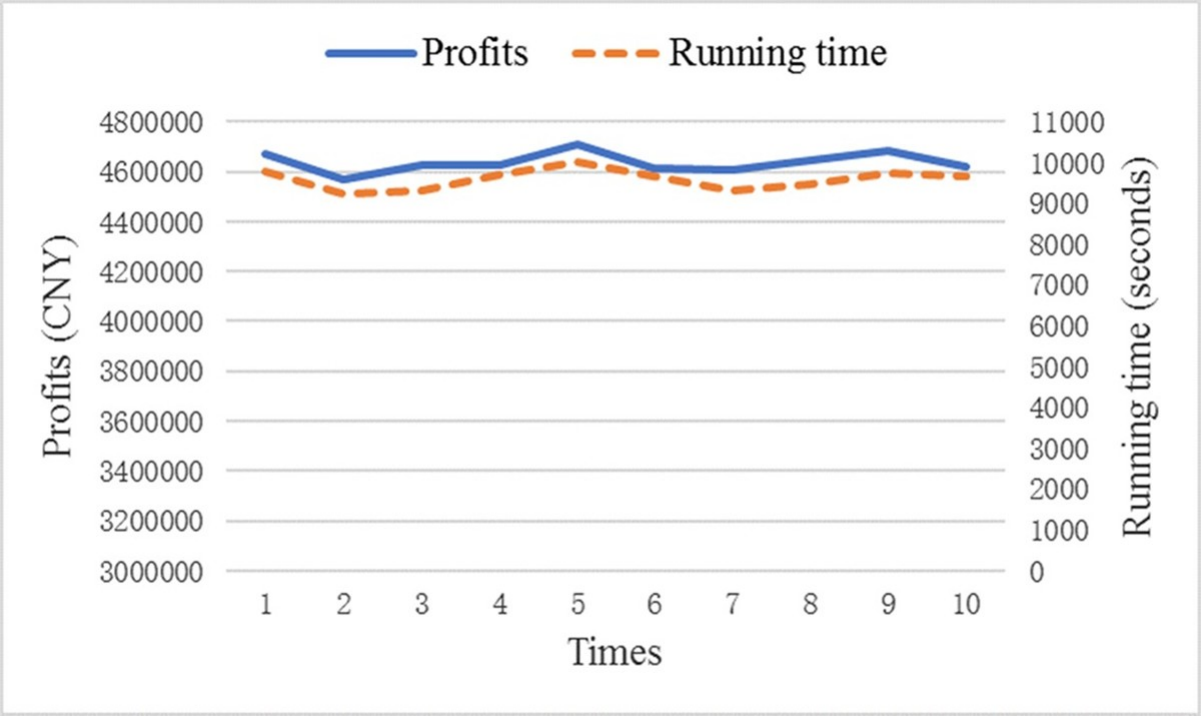
Results of IPSO.

The IPSO algorithm is developed in Python 3.5.3 (64bit) and independently run 10 times on a PC with a 2.60 GHz Intel CPU and 16.00 G RAM, under the Windows 8.1 operating system. The results of IPSO are shown in Fig 6. The maximum profits range from 4571233 CNY to 4712543 CNY (average, 4636853 CNY), and the running time is from 9253 seconds to 10023 seconds (average, 2 hours 39 minutes 53 seconds). Thus, the results show the robust stability of IPSO. The running time is acceptable because (1) The PSO has the ability to locate a good solution at a significantly faster rate than other evolutionary algorithms [32–35]; (2) Because the joint optimization of fleet sizing and empty pallet allocation is a strategic decision (long-term decision), the convergence speed of algorithms is not a critical issue for decision-makers.

The results show that *k*^5^ vehicles are selected. Decision-makers should configure eight, six, and six *k*^5^ vehicles for *i*^1^, *i*^2^, and *i*^3^, respectively. Although *k*^5^ can carry the fewest standard pallets in all types of vehicles, we find that *k*^5^ emits the lowest CO_2_ and its unitary transportation cost, idles cost, rental fee, and price are also the lowest. The optimization schemes are shown in Tables 5–9.

**Table 5:** Optimization scheme for period 1.

Station	*j*^1^	*j*^2^	*j*^3^	*j*^4^	*o*^1^	Rented vehicles	Idle vehicles
*i*^1^	2*	1	1	3	7	0	1
*i*^2^	1	2	1	1	5	0	1
*i*^3^	2	2	4	1	9	3	0

* Table 5 shows that 2, 1, 1, 3, and 7 k^5^ *vehicles are used to transport pallets from i*^1^ *to j*^1^, *from i*^1^ *to j*^2^, *from i*^1^ *to j*^3^, *from i*^1^ *to j*^4^, *and from o*^1^ *to i*^1^, *respectively*. *There is one idle vehicle at i*^1^ *and one idle vehicle at i*^2^. *i*^3^ *should rent three vehicles from vehicle rental companies. Tables 6–10 show information in the same way.*

**Table 6:** Optimization scheme for period 2.

Station	*j*^1^	*j*^2^	*j*^3^	*j*^4^	*o*^1^	Rented vehicles	Idle vehicles
*i*^1^	2	2	1	1	6	0	2
*i*^2^	1	1	1	3	6	0	0
*i*^3^	5	1	2	1	9	3	0

**Table 7:** Optimization scheme for period 3.

Station	*j*^1^	*j*^2^	*j*^3^	*j*^4^	*o*^1^	Rented vehicles	Idle vehicles
*i*^1^	3	2	2	1	8	0	0
*i*^2^	1	3	1	1	6	0	0
*i*^3^	2	1	1	2	6	0	0

**Table 8:** Optimization scheme for period 4.

Station	*j*^1^	*j*^2^	*j*^3^	*j*^4^	*o*^1^	Rented vehicles	Idle vehicles
*i*^1^	3	2	1	2	8	0	0
*i*^2^	2	2	1	1	6	0	0
*i*^3^	5	1	1	1	8	2	0

**Table 9:** Optimization scheme for period 5.

Station	*j*^1^	*j*^2^	*j*^3^	*j*^4^	*o*^1^	Rented vehicles	Idle vehicles
*i*^1^	2	2	1	3	8	0	0
*i*^2^	1	2	1	1	5	0	1
*i*^3^	2	1	1	2	6	0	0

## 4.2 Analysis

### 4.2.1 The effectiveness of IPSO

**Fig 7:**
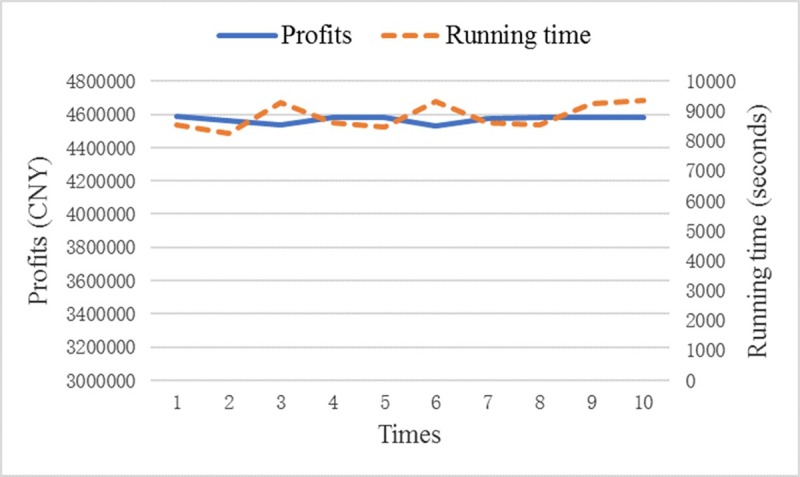
Results of CPSO.

We also apply another widely used modified PSO named the PSO with constriction coefficient (CPSO), which is proposed by Clerc and Kennedy [36], to solve the example presented in Subsection 4.1. To develop the CPSO algorithm in Python, we just need to use $v_{ij}^{n}=\lambda (v_{ij}^{o}+c1\times rnd_{1}\times (pbest_{ij}-p_{ij}^{o})+c2\times rnd_{2}\times (gbest_{j}-p_{ij}^{o}))$ instead of the velocity update equation in Step 4. $\lambda =\frac{2}{\left|2-\varphi -\sqrt{\varphi^{2}-4\varphi }\right|}$ is the constriction coefficient, where *φ* = *c*1+*c*2 and *c*1 = *c*2 = 2.05 [36]. We independently run the CPSO algorithm 10 times, and the results are shown in Fig 7. The average profits are 4570402 CNY and the average running time is 2 hours 27 minutes 3 seconds. Although the running time of CPSO is 12 minutes 50 seconds less than that of IPSO, the total profits obtained from CPSO are 66451 CNY less than those resulting from IPSO. Therefore, IPSO outperforms CPSO.

**Fig 8:**
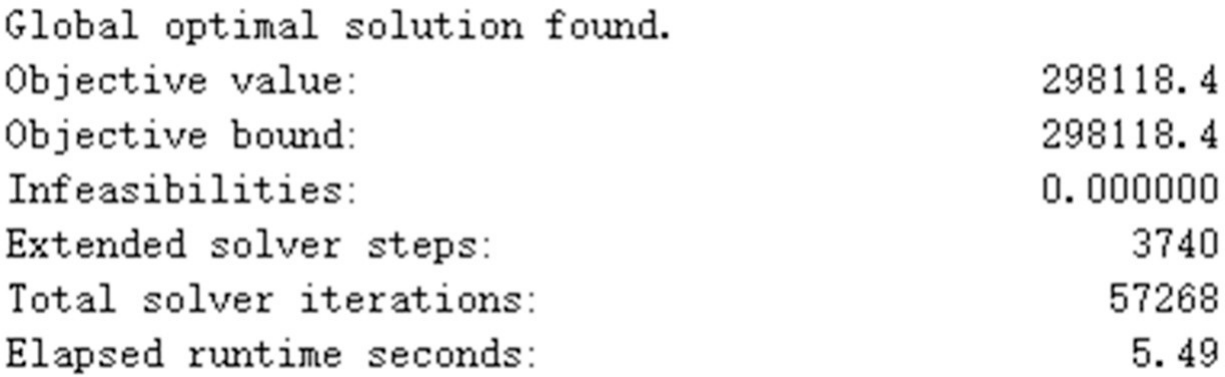
Results of Lingo.

To prove that IPSO can find global optima, we propose a simple case where the length of time periods is 1 (*t*^1^) and the pallet rental company only provides one kind of pallet (*p*^1^). The other parameters are the same as our example. Both Lingo software and IPSO are applied to solve the problem, and the resulting maximum profits are 298118 CNY and 295940 CNY, respectively. The total profits obtained from IPSO are only 2178 CNY (0.7%) less (lower) than those resulting from Lingo. Lingo uses the branch-and-bound method to solve integer models, and the results show that the globally optimal solution is found. Therefore, the optimal solution obtained from IPSO can be regarded as the global optimum. The results of Lingo are shown in Fig 8 and Table 10. Decision-makers should rent vehicles instead of buying them. *i*^1^, *i*^2^, and *i*^3^ should rent one *k*^2^, one *k*^5^, and two *k*^5^, respectively.

**Table 10:** Optimization scheme for the simple case.

Station	*j*^1^	*j*^2^	*j*^3^	*j*^4^	*o*^1^	Rented vehicles	Idle vehicles
***i***^1^	1	-	-	-	1	1	0
***i***^2^	-	-	-	1	1	1	0
***i***^3^	-	1	1	-	2	2	0

#### 4.2.2 Sensitivity analysis

**Fig 9:**
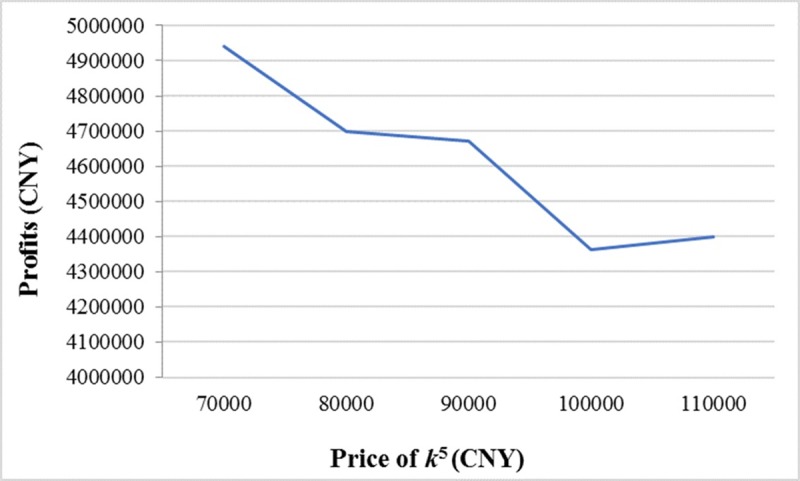
The relationship between expected total profits and the price of *k*^5^.

If the price of *k*^5^ is assigned the values of 70000 CNY, 80000 CNY, 90000 CNY, 100000 CNY, and 110000 CNY while the values of other parameters are the same as the proposed example in Subsection 4.1, the optimum vehicle types are(*k*^5^), (*k*^5^), (*k*^5^), (*k*^4^), and (*k*^4^), respectively. The expected total profits are shown in Fig 9. Therefore, managers would not choose *k*^5^ if it is too expensive, although the CO_2_ emissions from *k*^5^ are lower than *k*^4^.

**Fig 10:**
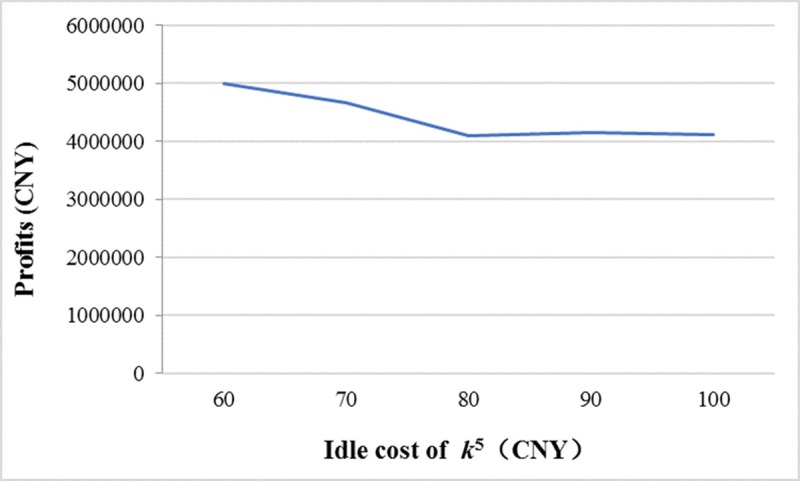
The relationship between expected total profits and the idle cost of *k*^5^.

If the idle cost of *k*^5^ is assigned the values of 60 CNY, 70 CNY, 80 CNY, 90 CNY, and 100 CNY while the values of other parameters are the same as the proposed example, the optimum vehicle types are(*k*^5^), (*k*^5^), (*k*^4^), (*k*^4^), and(*k*^4^), respectively. The expected total profits are shown in Fig 10. Although the CO_2_ emissions from *k*^5^ are lower than *k*^4^, managers would not choose *k*^5^ if its idle cost is too high. We also analyze the relationship between the expected total profits and the rental fee of *k*^5^, and find that managers would not choose *k*^5^ if the rental fee of *k*^5^ is too high.

## Conclusions

Pallet rental companies need to transport pallets from their pallet rental service stations (PRSSs) to customers and retrieve these pallets. Therefore, they should configure the right number and types of vehicles for PRSSs.

To address this problem, we propose an optimization model for fleet sizing and empty pallet allocation considering CO_2_ emissions. We take many factors into account to develop this model, e.g., uncertain demand, storage capacity, and rented vehicles. The objectives of this model are to maximize the profits of pallet rental companies and minimize CO_2_ emissions from vehicles because managers always pay attention to profits and should understand the environmental impacts of pallet logistics when they make decisions. A particle swarm optimization algorithm with inertia weight (IPSO) is developed in Python to solve the proposed model. IPSO can avoid falling into local optima because it can control the velocity by setting inertia factor, and it is able to find a globally optimal solution within a reasonable number of iterations. A numerical example is used to prove the effectiveness of the proposed approach. Two benchmarks algorithms, i.e., the PSO with constriction coefficient (CPSO) and the branch-and-bound method provided by Lingo, are used to examine the performance of IPSO. The results of numerical tests show that the amount of CO_2_ emissions from vehicles can affect the decision on fleet sizing and empty pallet allocation. However, the price, rental fees, and idle costs of the vehicles with low CO_2_ emissions cannot be too high.

To the best of our knowledge, this is the first study to propose a method for the joint optimization of fleet sizing and empty pallet allocation. The approach is useful for the managers of pallet rental companies because the fleet size can significantly affect the efficiency and costs of empty pallet allocation.

## Supporting information

S1 Code IPSO. (DOCX)

S2 Code Lingo. (DOCX)

S1 Table Mathematical notations. (DOCX)
