# Supplementary material for: An optimization model for fleet sizing and empty pallet allocation considering CO2 emissions
Source: PLoS One. 2020 Feb 21;15(2):e0229544. doi: 10.1371/journal.pone.0229544 (PMC7034922; doi:10.1371/journal.pone.0229544)
Supplement: S1 Code — (DOCX) [file pone.0229544.s001.docx]

**S1 Code. IPSO**

import numpy as np

import random

import matplotlib.pyplot as plt

import time

import matplotlib

import xlsxwriter

myfont =matplotlib.font_manager.FontProperties(fname='C:\Windows\Fonts\simsun.ttc')

np.set_printoptions(threshold=np.inf)

start = time.time()

r=5

#Price

FA=[400000,300000,200000,90000,90000]#[1,2,3,4,5]

#Distance

l=[[50,55,20],

[60,55,30],

[40,40,30],

[65,60,40],

[70,75,50]]#[i1,i2,i3]

# Turnover times

A=[[2,2,3],

[2,2,3],

[3,3,3],

[2,2,3],

[2,2,2]]

# Demands

B=[[2000,1600,1500,1200,6300],#t1-4p1 j1,j2,j3,j4,j5...j20

[2200,1600,1400,1300,6500],#t5-12p1 j1,j2,j3,j4,j5...j20

[2000+203,1500,1500,1300,6300+197],#t13-20p1 j1,j2,j3,j4,j5...j20

[2000,1500,1800,1800,7100],#t1-4p2 j1,j2,j3,j4,j5...j20

[3000,2000,1800,1800,8600],#t5-12p2 j1,j2,j3,j4,j5...j20

[3000+407,2000,1600,1800,8400+393]]#t13-20p2 j1,j2,j3,j4,j5...j20

# Transportation cost

CA=[0.75,0.6,0.55,0.4,0.4]#j20 i1i2i3

# Carrying capacity AAAAAAAA91000A7198708A92000A7161351A93000A 7037822A91500A7163718AAAAAAAAAAAAAAAAAAAAAAAAAAAAAAAAAAAAAAAAAAAAAAAAAAAAAAAAAAAAAAAAAAAAAAAAAAAAAAAAAAAAAAAAAAAAAAAAAAAAAAAAAAAAAAAAAAAAAAAAAAAA

DA=[400,300,200,120,120]#j20 1,2,3,4,5

# Storage capacity

K0=[60000,80000,80000]#[i1,i2,i3]

# The purchased pallets

NP=[[4000,4000,4000],#t1-4p1 i1i2i3

[3,3,3],#t5-12p1 i1i2i3

[23,23,23],#t13-20p1 i1i2i3

[4000,4000,4000],#t1-4p2 i1i2i3

[4,4,4],#t5-12p2 i1i2i3

[40,40,40]]#t13-20p2 i1i2i3

# Storage cost

pk1=[0.1,0.2,0.2]#p1[i1,i2,i3]

pk2=[0.12,0.4,0.4]#p2[i1,i2,i3]

# Loading and unloading cost

pl1=[0.12,0.12,0.12]#p1[i1,i2,i3]

pl2=[0.14,0.14,0.14]#p2[i1,i2,i3]

#MAX w, MIN w

wmin=0.4

wmax=0.9

# The unitary cost of CO2 emissions

gg=0.00004186

# CO_2_ emissions

hA=[598.03,514.03,501.64,326.88,175]

# Idle cost AAAAAAAAAAAAAAAAAAAAAAAAAAAAAAAAAAAAAAAAAAAAAAAAAAAAAAAAAAAAAAAAAAAAAAAAAAAAAAAAAAAAAAAAAAAAAAAAAAAAAAAAAAAAAAAAAAAAAAAAAAAAAAAAAAAAAAAAAAAAAAAAAAAAAAA

oA=[100,90,80,70,70]

# Rental fee AAAAAAAAAAAAA33000A7244206AAAAAAAAAAAAAAAAAAAAAAAAAAAAAAAAAAAAAAAAAAAAAAAAAAAAAAAAAAAAAAAAAAAAAAAAAAAAAAAAAAAAAAAAAAAAAAAAAAAAAAAAAAAAAAAAAAAAAAAAAAAAAAAAAAAAAAAAAA

pA=[80000,60000,50000,30000,30000]

class PSO1():

def __init__(self, pN, dim, max_iter):

self.w = 2 #Certain value

self.c1 = 2

self.c2 = 2

self.r1 = 2

self.r2 = 2

self.pN = pN # Number of particles

self.dim = dim # Search dimension

self.max_iter = max_iter # Number of iterations

self.X = np.zeros((self.pN, self.dim)) # Position and velocity

self.V = np.zeros((self.pN, self.dim))

self.pbest = np.zeros((self.pN, self.dim)) # Pbest

self.gbest = np.zeros((1, self.dim))

self.p_fit = np.zeros(self.pN) # Fitness

self.fit = -9999999999999999999999999

#Objective function

def function(self, X):#X=[p1 t1 j1 i1,p1 t1 j1 i2,p1 t1 j1 i3,p1 t1 j2 i1...p1 t2 j1 i1...p2 t1 j1 i1]

def fitness1(X):

ss=np.array(X)

s=0

for i in range(150):

if(i+3)%15==0:

continue

elif(i+2)%15==0:

continue

elif (i+1)%15==0:

continue

else:

if i<75:

s+=ss[i]

else: s+=ss[i]

return s

def fitness2(X):

s=0

for i in range(3):

if i<3:

s+=X[i+225]*F[i//3]

return s

def fitness34(X):

s=X

l1=[]

A1=[]

sum=0

for i in range(5):

l1+=l[i]

A1+=A[i]

e=l1*5

f=A1*5

for i in range(75):

sum+=r*e[i]*s[i+150]*f[i]*C[i//75]

return sum

def fitness5(X):

s=X

sum1=0

sum2=0

sum3=0

sum4=0

sum5=0

sum6=0

d1=np.zeros(10)

d2=np.zeros(10)

d3=np.zeros(10)

e=[]

g=[]

h=[]

i=[]

for i in range(150):

if(i+1)%15==0:

d3[(i+1)//15-1]=s[i]-s[i-3]-s[i-6]-s[i-9]-s[i-12]#3

d2[(i+1)//15-1]=s[i-1]-s[i-4]-s[i-7]-s[i-10]-s[i-13]#2

d1[(i+1)//15-1]=s[i-2]-s[i-5]-s[i-8]-s[i-11]-s[i-14]#1

for i in range(30):

if i%3==0:

e.append(d1[i//3])

elif i%3==1:

e.append(d2[i//3])

else: e.append(d3[i//3])#e xo-xi[p1 t1 i1, p1 t1 i2]

g=NP[0]+NP[1]*2+NP[2]*2+NP[3]+NP[4]*2+NP[5]*2

f=np.array(e)+np.array(g)# +xo-xi

for m in range(15):

if m%3==0:

sum1+=f[m]

h.append(sum1)

elif m%3==1:

sum2+=f[m]

h.append(sum2)

else:

sum3+=f[m]

h.append(sum3)

for m in range(15,30):

if m%3==0:

sum4+=f[m]

h.append(sum4)

elif m%3==1:

sum5+=f[m]

h.append(sum5)

else:

sum6+=f[m]

h.append(sum6)

sum=0

i=pk1*5+pk2*5

for m in range(30):

sum+=np.array(h[m])*np.array(i[m])

return sum

def fitness67(X):

pl=pl1*25+pl2*25

s=X

sum=0

for i in range(150):

sum+=s[i]*pl[i]

return sum

def fitness89(X):

s=np.array(X)

sum=0

d=np.zeros(15)#vijkt

e=np.zeros(15)#vik

f=np.zeros(15)#xianzhi

g=np.zeros(15)#zu

a=0

b=0

c=0

for i in range(75):

if i%15==0:

d[(i//15)*3]=s[150+i]+s[153+i]+s[156+i]+s[159+i]

d[(i//15)*3+1]=s[151+i]+s[154+i]+s[157+i]+s[160+i]

d[(i//15)*3+2]=s[152+i]+s[155+i]+s[158+i]+s[161+i]

for i in range(1):

e[15*i+0]=s[i*3+225]

e[15*i+1]=s[i*3+226]

e[15*i+2]=s[i*3+227]

e[15*i+3]=s[i*3+225]

e[15*i+4]=s[i*3+226]

e[15*i+5]=s[i*3+227]

e[15*i+6]=s[i*3+225]

e[15*i+7]=s[i*3+226]

e[15*i+8]=s[i*3+227]

e[15*i+9]=s[i*3+225]

e[15*i+10]=s[i*3+226]

e[15*i+11]=s[i*3+227]

e[15*i+12]=s[i*3+225]

e[15*i+13]=s[i*3+226]

e[15*i+14]=s[i*3+227]

for i in range(15):

if i <15:

f[i]=o[0]

elif i<30:

f[i]=o[1]

elif i<45:

f[i]=o[2]

elif i<60:

f[i]=o[3]

else:

f[i]=o[4]

for i in range(15):

if i <15:

g[i]=p[0]

elif i<30:

g[i]=p[1]

elif i<45:

g[i]=p[2]

elif i<60:

g[i]=p[3]

else:

g[i]=p[4]

for i in range(15):

if e[i]>d[i]:

sum+=f[i]*(e[i]-d[i])

else:

sum+=g[i]*(d[i]-e[i])

return sum

def fitness10(X):

s=X

l1=[]

A1=[]

sum=0

for i in range(5):

l1+=l[i]

A1+=A[i]

e=l1*5

f=A1*5

for i in range(75):

sum+=r*e[i]*s[i+150]*f[i]*gg*h[i//75]

return sum

def T35(X):

c=0

s=[]

b=B[0]+B[1]*2+B[2]*2+B[3]+B[4]*2+B[5]*2

for i in range(0,150):

if i%3==0:

s.append(X[i]+X[i+1]+X[i+2])

for i in range(0,50):

if (i+1)%5==0:

if s[i]>=b[i]:

c+=0

else:c+=-120*(b[i]-s[i])

else:

if s[i]<=b[i]:

c+=0

else:c+=-120*(s[i]-b[i])

return c

def T46(X):

s=X

A1=[]

g=np.zeros(75)

for i in range(5):

A1+=A[i]

f=A1*5

c=0

d=np.zeros(75)

e=np.zeros(75)

for i in range(75):

d[i]=D[0]

for i in range(75):

e[i]=r*(d[i]*f[i]*s[i+150])

for i in range(75):

g[i]=s[i]+s[i+75]*1.1

for i in range(75):

if e[i]>=g[i]:

c+=0

else:

c+=-700000*(g[i]-e[i])

#AAAAAAAAAAAAAAAAAAAAAAAAAAAAAAAAAAAAAAAAAAAAAAAAAAAAAAAAAAAAAAAAAAAAAAAAAAAAAAAAAAAAAAAAAAAAAAAAAAAAAAAAAAAAAAAAAAAAAAAAAAAAAAAAAAAAAAAAAAAAAAAAAAAAAAA

return c

def T79(X):

s=X

sum1=0

sum2=0

sum3=0

sum4=0

sum5=0

sum6=0

d1=np.zeros(10)

d2=np.zeros(10)

d3=np.zeros(10)

e=[]

g=[]

h=[]

i=[]

k1=np.zeros(10)

k2=np.zeros(10)

k3=np.zeros(10)

l=[]

for i in range(150):

if(i+1)%15==0:

d3[(i+1)//15-1]=s[i]-s[i-3]-s[i-6]-s[i-9]-s[i-12]

d2[(i+1)//15-1]=s[i-1]-s[i-4]-s[i-7]-s[i-10]-s[i-13]

d1[(i+1)//15-1]=s[i-2]-s[i-5]-s[i-8]-s[i-11]-s[i-14]

for i in range(30):

if i%3==0:

e.append(d1[i//3])

elif i%3==1:

e.append(d2[i//3])

else: e.append(d3[i//3])

g=NP[0]+NP[1]*2+NP[2]*2+NP[3]+NP[4]*2+NP[5]*2

f=np.array(e)+np.array(g)

for m in range(15):

if m%3==0:

sum1+=f[m]

h.append(sum1)

elif m%3==1:

sum2+=f[m]

h.append(sum2)

else:

sum3+=f[m]

h.append(sum3)

for m in range(15,30):

if m%3==0:

sum4+=f[m]

h.append(sum4)

elif m%3==1:

sum5+=f[m]

h.append(sum5)

else:

sum6+=f[m]

h.append(sum6)

for i in range(150):

if(i+1)%15==0:

k3[(i+1)//15-1]=s[i-3]+s[i-6]+s[i-9]+s[i-12]

k2[(i+1)//15-1]=s[i-4]+s[i-7]+s[i-10]+s[i-13]

k1[(i+1)//15-1]=s[i-5]+s[i-8]+s[i-11]+s[i-14]

for i in range(30):

if i%3==0:

l.append(k1[i//3])

elif i%3==1:

l.append(k2[i//3])

else: l.append(k3[i//3])

z=0

y=0

for i in range(30):

if ((i>=0)and(i<=2))or((i>=15)and(i<=17)):

if l[i]<=g[i]:

z+=0

else:z+=-120*(l[i]-g[i])

else :

if l[i]<=g[i]+h[i-3]:

z+=0

else:z+=-120*(l[i]-g[i]-h[i-3])

o=0

for i in range(15):

if i%3==0:

o=0

elif i%3==1:

o=1

else:

o=2

if 1.1*h[i+15]+h[i]<=K0[o]:

y+=0

else:y+=-120*(1.1*h[i+15]+h[i]-K0[o])

return z+y

s=fitness1(X)-fitness2(X)-fitness34(X)-fitness5(X)-fitness67(X)-fitness89(X)-fitness10(X)+T35(X)+T46(X)+T79(X)

return s

# Randomly generate a population of particles

def init_Population(self):

b=B[0]+B[1]*2+B[2]*2+B[3]+B[4]*2+B[5]*2

for i in range(self.pN):

for j in range(self.dim):

if j <150:

self.V[i][j] = random.uniform(-5, 5)

if j%3==0:

self.X[i][j] = round(random.randint(0, b[j//3]))

self.X[i][j+1] = round(random.randint(0, b[j//3]-self.X[i][j]))

self.X[i][j+2] =round(b[j//3]-self.X[i][j]-self.X[i][j+1])

elif j<225:

self.X[i][j]=0

self.V[i][j] = random.uniform(-5,5)

else:

self.V[i][j] = random.uniform(-5, 5)

#####################################################################################

################

self.X[i][225]=0

self.X[i][226]=0

self.X[i][227]=0

self.pbest[i] = self.X[i]

tmp = self.function(self.X[i])

self.p_fit[i] = tmp

if tmp > self.fit:

self.fit = tmp

self.gbest = self.X[i]

#Update position

def iterator(self):

for t in range(self.max_iter):

self.w=wmin + (wmax - wmin)*(self.max_iter - t)/self.max_iter

c1s = 2.5

c1d = 0.5

c2s = 0.5

c2d = 2.5

self.c1 = (c1s - c1d)*(self.max_iter - t)/self.max_iter + c1d

self.c2 = (c2s - c2d)*(self.max_iter - t)/self.max_iter + c2d

for i in range(self.pN): # Update gbest\pbest

temp = self.function(self.X[i])

if temp > self.p_fit[i]: # Update pbest

self.p_fit[i] = temp

self.pbest[i] = self.X[i]

if self.p_fit[i] > self.fit: # Update gbest

self.gbest = self.X[i]

self.fit = self.p_fit[i]

for k in range(self.dim):

self.r1=random.random()

self.r2=random.random()

self.V[i][k] = self.w * self.V[i][k] + self.c1 * self.r1 * (self.pbest[i][k] - self.X[i][k]) + self.c2 * self.r2 * (self.gbest[k] - self.X[i][k])#Update velocity

# velocity

if k <150:

# velocity

if self.V[i][k]<-10:

self.V[i][k] = -10

if self.V[i][k]>10:

self.V[i][k] = 10

else:

if self.V[i][k]<-10:

self.V[i][k] = -10

if self.V[i][k]>10:

self.V[i][k] = 10

self.X[i] = np.round(self.X[i] + self.V[i])#Update position

self.X[i] =abs(self.X[i])

fitness.append(np.round(self.fit))

print(self.gbest//1, end=" ")

print(self.fit//1)# Output optimal value

#####################################################################################

print(self.function(self.X[1])//1)

if t==(self.max_iter-1):

for i in range(228):

QA.append(self.gbest[i])

return fitness

#Program

Fitness1=[]

QA1=[]

for i in range(5):#1

QA=[]

F=[]#[1,2,3,4,5]

#Distance

#[i1,i2,i3]

#Turnover times

fitness=[]

# Transportation cost

C=[]#12345

# Carrying capacity

D=[]#j20 1,2,3,4,5

# Storage cost

#p1[i1,i2,i3]

#p2[i1,i2,i3]

#Loading and unloading cost

#p1[i1,i2,i3]

#p2[i1,i2,i3]

#CO2 g/km

h=[]

#Idle cost

o=[]

#Rental fee

p=[]

F.append(FA[i])

C.append(CA[i])

D.append(DA[i])

h.append(hA[i])

o.append(oA[i])

p.append(pA[i])

my_pso = PSO1(pN=80, dim=228, max_iter=2000)#

my_pso.init_Population()

fitness = my_pso.iterator()

Fitness1.append(fitness)

QA1.append(QA)

class PSO2():

def __init__(self, pN, dim, max_iter):

self.w = 2 #

self.c1 = 2

self.c2 = 2

self.r1 = 2

self.r2 = 2

self.pN = pN # Number of particles

self.dim = dim # Search dimension

self.max_iter = max_iter # Number of iterations

self.X = np.zeros((self.pN, self.dim)) # Position and velocity

self.V = np.zeros((self.pN, self.dim))

self.pbest = np.zeros((self.pN, self.dim)) # Update pbest and gbest

self.gbest = np.zeros((1, self.dim))

self.p_fit = np.zeros(self.pN) # pbest

self.fit = -9999999999999999999999999

#Objective function

def function(self, X):#X=[p1 t1 j1 i1,p1 t1 j1 i2,p1 t1 j1 i3,p1 t1 j2 i1...p1 t2 j1 i1...p2 t1 j1 i1]

def fitness1(X):

ss=np.array(X)

s=0

for i in range(150):

if(i+3)%15==0:

continue

elif(i+2)%15==0:

continue

elif (i+1)%15==0:

continue

else:

if i<75:

s+=ss[i]*72

else: s+=ss[i]*108

return s

def fitness2(X):

s=0

for i in range(6):

if i<3:

s+=X[i+300]*F[i//3]

elif i<6:

s+=X[i+300]*F[i//3]

elif i<9:

s+=X[i+300]*F[i//3]

elif i<12:

s+=X[i+300]*F[i//3]

else:

s+=X[i+300]*F[i//3]

return s

def fitness34(X):

s=X

l1=[]

A1=[]

sum=0

for i in range(5):

l1+=l[i]

A1+=A[i]

e=l1*10

f=A1*10

for i in range(150):

sum+=r*e[i]*s[i+150]*f[i]*C[i//75]

return sum

def fitness5(X):

s=X

sum1=0

sum2=0

sum3=0

sum4=0

sum5=0

sum6=0

d1=np.zeros(10)

d2=np.zeros(10)

d3=np.zeros(10)

e=[]

g=[]

h=[]

i=[]

for i in range(150):

if(i+1)%15==0:

d3[(i+1)//15-1]=s[i]-s[i-3]-s[i-6]-s[i-9]-s[i-12]#3

d2[(i+1)//15-1]=s[i-1]-s[i-4]-s[i-7]-s[i-10]-s[i-13]#2

d1[(i+1)//15-1]=s[i-2]-s[i-5]-s[i-8]-s[i-11]-s[i-14]#1

for i in range(30):

if i%3==0:

e.append(d1[i//3])

elif i%3==1:

e.append(d2[i//3])

else: e.append(d3[i//3])#e xo-xi[p1 t1 i1, p1 t1 i2]

g=NP[0]+NP[1]*2+NP[2]*2+NP[3]+NP[4]*2+NP[5]*2#the purchased pallets

f=np.array(e)+np.array(g)# +xo-xi

for m in range(15):

if m%3==0:

sum1+=f[m]

h.append(sum1)

elif m%3==1:

sum2+=f[m]

h.append(sum2)

else:

sum3+=f[m]

h.append(sum3)

for m in range(15,30):

if m%3==0:

sum4+=f[m]

h.append(sum4)

elif m%3==1:

sum5+=f[m]

h.append(sum5)

else:

sum6+=f[m]

h.append(sum6)

sum=0

i=pk1*5+pk2*5

for m in range(30):

sum+=np.array(h[m])*np.array(i[m])

return sum

def fitness67(X):

pl=pl1*25+pl2*25

s=X

sum=0

for i in range(150):

sum+=s[i]*pl[i]

return sum

def fitness89(X):

s=np.array(X)

sum=0

d=np.zeros(30)#vijkt

e=np.zeros(30)#vik

f=np.zeros(30)#xianzhi

g=np.zeros(30)#zu

a=0

b=0

c=0

for i in range(150):

if i%15==0:

d[(i//15)*3]=s[150+i]+s[153+i]+s[156+i]+s[159+i]

d[(i//15)*3+1]=s[151+i]+s[154+i]+s[157+i]+s[160+i]

d[(i//15)*3+2]=s[152+i]+s[155+i]+s[158+i]+s[161+i]

for i in range(2):

e[15*i+0]=s[i*3+300]

e[15*i+1]=s[i*3+301]

e[15*i+2]=s[i*3+302]

e[15*i+3]=s[i*3+300]

e[15*i+4]=s[i*3+301]

e[15*i+5]=s[i*3+302]

e[15*i+6]=s[i*3+300]

e[15*i+7]=s[i*3+301]

e[15*i+8]=s[i*3+302]

e[15*i+9]=s[i*3+300]

e[15*i+10]=s[i*3+301]

e[15*i+11]=s[i*3+302]

e[15*i+12]=s[i*3+300]

e[15*i+13]=s[i*3+301]

e[15*i+14]=s[i*3+302]

for i in range(30):

if i <15:

f[i]=o[0]

elif i<30:

f[i]=o[1]

elif i<45:

f[i]=o[2]

elif i<60:

f[i]=o[3]

else:

f[i]=o[4]

for i in range(30):

if i <15:

g[i]=p[0]

elif i<30:

g[i]=p[1]

elif i<45:

g[i]=p[2]

elif i<60:

g[i]=p[3]

else:

g[i]=p[4]

for i in range(30):

if e[i]>d[i]:

sum+=f[i]*(e[i]-d[i])

else:

sum+=g[i]*(d[i]-e[i])

return sum

def fitness10(X):

s=X

l1=[]

A1=[]

sum=0

for i in range(5):

l1+=l[i]

A1+=A[i]

e=l1*10

f=A1*10

for i in range(150):

sum+=r*e[i]*s[i+150]*f[i]*gg*h[i//75]

return sum

def T35(X):

c=0

s=[]

b=B[0]+B[1]*2+B[2]*2+B[3]+B[4]*2+B[5]*2

for i in range(0,150):

if i%3==0:

s.append(X[i]+X[i+1]+X[i+2])

for i in range(0,50):

if (i+1)%5==0:

if s[i]>=b[i]:

c+=0

else:c+=-120*(b[i]-s[i])

else:

if s[i]<=b[i]:

c+=0

else:c+=-120*(s[i]-b[i])

return c

def T46(X):

s=X

A1=[]

g=np.zeros(75)

for i in range(5):

A1+=A[i]

f=A1*10

c=0

d=np.zeros(150)

e=np.zeros(75)

for i in range(75):

d[i]=D[0]

for i in range(75,150):

d[i]=D[1]

for i in range(75):

e[i]=r*(d[i]*f[i]*s[i+150]+d[i+75]*f[i+75]*s[i+225])

for i in range(75):

g[i]=s[i]+s[i+75]*1.1

for i in range(75):

if e[i]>=g[i]:

c+=0

else:

c+=-700000*(g[i]-e[i])

#AAAAAAAAAAAAAAAAAAAAAAAAAAAAAAAAAAAAAAAAAAAAAAAAAAAAAAAAAAAAAAAAAAAAAAAAAAAAAAAAAAAAAAAAAAAAAAAAAAAAAAAAAAAAAAAAAAAAAAAAAAAAAAAAAAAAAAAAAAAAAAAAAAAAAAA

return c

def T79(X):

s=X

sum1=0

sum2=0

sum3=0

sum4=0

sum5=0

sum6=0

d1=np.zeros(10)

d2=np.zeros(10)

d3=np.zeros(10)

e=[]

g=[]

h=[]

i=[]

k1=np.zeros(10)

k2=np.zeros(10)

k3=np.zeros(10)

l=[]

for i in range(150):

if(i+1)%15==0:

d3[(i+1)//15-1]=s[i]-s[i-3]-s[i-6]-s[i-9]-s[i-12]

d2[(i+1)//15-1]=s[i-1]-s[i-4]-s[i-7]-s[i-10]-s[i-13]

d1[(i+1)//15-1]=s[i-2]-s[i-5]-s[i-8]-s[i-11]-s[i-14]

for i in range(30):

if i%3==0:

e.append(d1[i//3])

elif i%3==1:

e.append(d2[i//3])

else: e.append(d3[i//3])

g=NP[0]+NP[1]*2+NP[2]*2+NP[3]+NP[4]*2+NP[5]*2

f=np.array(e)+np.array(g)

for m in range(15):

if m%3==0:

sum1+=f[m]

h.append(sum1)

elif m%3==1:

sum2+=f[m]

h.append(sum2)

else:

sum3+=f[m]

h.append(sum3)

for m in range(15,30):

if m%3==0:

sum4+=f[m]

h.append(sum4)

elif m%3==1:

sum5+=f[m]

h.append(sum5)

else:

sum6+=f[m]

h.append(sum6)

for i in range(150):

if(i+1)%15==0:

k3[(i+1)//15-1]=s[i-3]+s[i-6]+s[i-9]+s[i-12]

k2[(i+1)//15-1]=s[i-4]+s[i-7]+s[i-10]+s[i-13]

k1[(i+1)//15-1]=s[i-5]+s[i-8]+s[i-11]+s[i-14]

for i in range(30):

if i%3==0:

l.append(k1[i//3])

elif i%3==1:

l.append(k2[i//3])

else: l.append(k3[i//3])

z=0

y=0

for i in range(30):

if ((i>=0)and(i<=2))or((i>=15)and(i<=17)):

if l[i]<=g[i]:

z+=0

else:z+=-120*(l[i]-g[i])

else :

if l[i]<=g[i]+h[i-3]:

z+=0

else:z+=-120*(l[i]-g[i]-h[i-3])

o=0

for i in range(15):

if i%3==0:

o=0

elif i%3==1:

o=1

else:

o=2

if 1.1*h[i+15]+h[i]<=K0[o]:

y+=0

else:y+=-120*(1.1*h[i+15]+h[i]-K0[o])

return z+y

s=fitness1(X)-fitness2(X)-fitness34(X)-fitness5(X)-fitness67(X)-fitness89(X)-fitness10(X)+T35(X)+T46(X)+T79(X)

return s

#initialization

def init_Population(self):

b=B[0]+B[1]*2+B[2]*2+B[3]+B[4]*2+B[5]*2

for i in range(self.pN):

for j in range(self.dim):

if j <150:

self.V[i][j] = random.uniform(-5, 5)

if j%3==0:

self.X[i][j] = round(random.randint(0, b[j//3]))

self.X[i][j+1] = round(random.randint(0, b[j//3]-self.X[i][j]))

self.X[i][j+2] =round(b[j//3]-self.X[i][j]-self.X[i][j+1])

elif j<225:

self.X[i][j]=0

self.X[i][j+75]=0

self.V[i][j] = random.uniform(-5, 5)

self.V[i][j+75] = random.uniform(-5, 5)

else:

self.V[i][j] = random.uniform(-5, 5)

#####################################################################################

################

self.X[i][300]=0

self.X[i][301]=0

self.X[i][302]=0

self.X[i][303]=0

self.X[i][304]=0

self.X[i][305]=0

self.pbest[i] = self.X[i]

tmp = self.function(self.X[i])

self.p_fit[i] = tmp

if tmp > self.fit:

self.fit = tmp

self.gbest = self.X[i]

#Update position

def iterator(self):

for t in range(self.max_iter):

self.w=wmin + (wmax - wmin)*(self.max_iter - t)/self.max_iter

c1s = 2.5

c1d = 0.5

c2s = 0.5

c2d = 2.5

self.c1 = (c1s - c1d)*(self.max_iter - t)/self.max_iter + c1d

self.c2 = (c2s - c2d)*(self.max_iter - t)/self.max_iter + c2d

for i in range(self.pN): # Update gbest\pbest

temp = self.function(self.X[i])

if temp > self.p_fit[i]: # Update pbest

self.p_fit[i] = temp

self.pbest[i] = self.X[i]

if self.p_fit[i] > self.fit: # Update gbest

self.gbest = self.X[i]

self.fit = self.p_fit[i]

for k in range(self.dim):

self.r1=random.random()

self.r2=random.random()

self.V[i][k] = self.w * self.V[i][k] + self.c1 * self.r1 * (self.pbest[i][k] - self.X[i][k]) + self.c2 * self.r2 * (self.gbest[k] - self.X[i][k])#Update velocity

# velocity

if k <150:

# velocity

if self.V[i][k]<-10:

self.V[i][k] = -10

if self.V[i][k]>10:

self.V[i][k] = 10

else:

if self.V[i][k]<-10:

self.V[i][k] = -10

if self.V[i][k]>10:

self.V[i][k] = 10

self.X[i] = np.round(self.X[i] + self.V[i])#Update position

self.X[i] =abs(self.X[i])

fitness.append(np.round(self.fit))

print(self.gbest//1, end=" ")

print(self.fit//1)# Output optimal value

#####################################################################################

print(self.function(self.X[1])//1)

if t==(self.max_iter-1):

for i in range(306):

QA.append(self.gbest[i])

return fitness

#Program

Fitness2=[]

QA2=[]

for i in range(4):#1

for j in range(i+1,5):#2

QA=[]

F=[]#[1,2,3,4,5]

#Distance

#[i1,i2,i3]

#Turnover times

fitness=[]

# Transportation cost

C=[]#12345

# Carrying capacity

D=[]#j20 1,2,3,4,5

# Storage cost

#p1[i1,i2,i3]

#p2[i1,i2,i3]

# Loading and unloading cost

#p1[i1,i2,i3]

#p2[i1,i2,i3]

#CO2 g/km

h=[]

#Idle cost

o=[]

#Rental fee

p=[]

F.append(FA[i])

C.append(CA[i])

D.append(DA[i])

h.append(hA[i])

o.append(oA[i])

p.append(pA[i])

F.append(FA[j])

C.append(CA[j])

D.append(DA[j])

h.append(hA[j])

o.append(oA[j])

p.append(pA[j])

my_pso = PSO2(pN=80, dim=306, max_iter=2000)#

my_pso.init_Population()

fitness = my_pso.iterator()

Fitness2.append(fitness)

QA2.append(QA)

class PSO3():

def __init__(self, pN, dim, max_iter):

self.w = 2

self.c1 = 2

self.c2 = 2

self.r1 = 2

self.r2 = 2

self.pN = pN # Number of particles

self.dim = dim # Search dimension

self.max_iter = max_iter # Number of iterations

self.X = np.zeros((self.pN, self.dim)) # position and velocity

self.V = np.zeros((self.pN, self.dim))

self.pbest = np.zeros((self.pN, self.dim)) # pbest and gbest

self.gbest = np.zeros((1, self.dim))

self.p_fit = np.zeros(self.pN) # pbest

self.fit = -9999999999999999999999999

#Objective funtion

def function(self, X):#X=[p1 t1 j1 i1,p1 t1 j1 i2,p1 t1 j1 i3,p1 t1 j2 i1...p1 t2 j1 i1...p2 t1 j1 i1]

def fitness1(X):

ss=np.array(X)

s=0

for i in range(150):

if(i+3)%15==0:

continue

elif(i+2)%15==0:

continue

elif (i+1)%15==0:

continue

else:

if i<75:

s+=ss[i]*72

else: s+=ss[i]*108

return s

def fitness2(X):

s=0

for i in range(9):

if i<3:

s+=X[i+375]*F[i//3]

elif i<6:

s+=X[i+375]*F[i//3]

elif i<9:

s+=X[i+375]*F[i//3]

elif i<12:

s+=X[i+375]*F[i//3]

else:

s+=X[i+375]*F[i//3]

return s

def fitness34(X):

s=X

l1=[]

A1=[]

sum=0

for i in range(5):

l1+=l[i]

A1+=A[i]

e=l1*15

f=A1*15

for i in range(225):

sum+=r*e[i]*s[i+150]*f[i]*C[i//75]

return sum

def fitness5(X):

s=X

sum1=0

sum2=0

sum3=0

sum4=0

sum5=0

sum6=0

d1=np.zeros(10)

d2=np.zeros(10)

d3=np.zeros(10)

e=[]

g=[]

h=[]

i=[]

for i in range(150):

if(i+1)%15==0:

d3[(i+1)//15-1]=s[i]-s[i-3]-s[i-6]-s[i-9]-s[i-12]#3

d2[(i+1)//15-1]=s[i-1]-s[i-4]-s[i-7]-s[i-10]-s[i-13]#2

d1[(i+1)//15-1]=s[i-2]-s[i-5]-s[i-8]-s[i-11]-s[i-14]#1

for i in range(30):

if i%3==0:

e.append(d1[i//3])

elif i%3==1:

e.append(d2[i//3])

else: e.append(d3[i//3])#e xo-xi[p1 t1 i1, p1 t1 i2]

g=NP[0]+NP[1]*2+NP[2]*2+NP[3]+NP[4]*2+NP[5]*2

f=np.array(e)+np.array(g)# +xo-xi

for m in range(15):

if m%3==0:

sum1+=f[m]

h.append(sum1)

elif m%3==1:

sum2+=f[m]

h.append(sum2)

else:

sum3+=f[m]

h.append(sum3)

for m in range(15,30):

if m%3==0:

sum4+=f[m]

h.append(sum4)

elif m%3==1:

sum5+=f[m]

h.append(sum5)

else:

sum6+=f[m]

h.append(sum6)

sum=0

i=pk1*5+pk2*5

for m in range(30):

sum+=np.array(h[m])*np.array(i[m])

return sum

def fitness67(X):

pl=pl1*25+pl2*25

s=X

sum=0

for i in range(150):

sum+=s[i]*pl[i]

return sum

def fitness89(X):

s=np.array(X)

sum=0

d=np.zeros(45)#vijkt

e=np.zeros(45)#vik

f=np.zeros(45)#xianzhi

g=np.zeros(45)#zu

a=0

b=0

c=0

for i in range(225):

if i%15==0:

d[(i//15)*3]=s[150+i]+s[153+i]+s[156+i]+s[159+i]

d[(i//15)*3+1]=s[151+i]+s[154+i]+s[157+i]+s[160+i]

d[(i//15)*3+2]=s[152+i]+s[155+i]+s[158+i]+s[161+i]

for i in range(3):

e[15*i+0]=s[i*3+375]

e[15*i+1]=s[i*3+376]

e[15*i+2]=s[i*3+377]

e[15*i+3]=s[i*3+375]

e[15*i+4]=s[i*3+376]

e[15*i+5]=s[i*3+377]

e[15*i+6]=s[i*3+375]

e[15*i+7]=s[i*3+376]

e[15*i+8]=s[i*3+377]

e[15*i+9]=s[i*3+375]

e[15*i+10]=s[i*3+376]

e[15*i+11]=s[i*3+377]

e[15*i+12]=s[i*3+375]

e[15*i+13]=s[i*3+376]

e[15*i+14]=s[i*3+377]

for i in range(45):

if i <15:

f[i]=o[0]

elif i<30:

f[i]=o[1]

elif i<45:

f[i]=o[2]

elif i<60:

f[i]=o[3]

else:

f[i]=o[4]

for i in range(45):

if i <15:

g[i]=p[0]

elif i<30:

g[i]=p[1]

elif i<45:

g[i]=p[2]

elif i<60:

g[i]=p[3]

else:

g[i]=p[4]

for i in range(45):

if e[i]>d[i]:

sum+=f[i]*(e[i]-d[i])

else:

sum+=g[i]*(d[i]-e[i])

return sum

def fitness10(X):

s=X

l1=[]

A1=[]

sum=0

for i in range(5):

l1+=l[i]

A1+=A[i]

e=l1*15

f=A1*15

for i in range(225):

sum+=r*e[i]*s[i+150]*f[i]*gg*h[i//75]

return sum

def T35(X):

c=0

s=[]

b=B[0]+B[1]*2+B[2]*2+B[3]+B[4]*2+B[5]*2

for i in range(0,150):

if i%3==0:

s.append(X[i]+X[i+1]+X[i+2])

for i in range(0,50):

if (i+1)%5==0:

if s[i]>=b[i]:

c+=0

else:c+=-120*(b[i]-s[i])

else:

if s[i]<=b[i]:

c+=0

else:c+=-120*(s[i]-b[i])

return c

def T46(X):

s=X

A1=[]

g=np.zeros(75)

for i in range(5):

A1+=A[i]

f=A1*15

c=0

d=np.zeros(225)

e=np.zeros(75)

for i in range(75):

d[i]=D[0]

for i in range(75,150):

d[i]=D[1]

for i in range(150,225):

d[i]=D[2]

for i in range(75):

e[i]=r*(d[i]*f[i]*s[i+150]+d[i+75]*f[i+75]*s[i+225]+d[i+150]*f[i+150]*s[i+300])

for i in range(75):

g[i]=s[i]+s[i+75]*1.1

for i in range(75):

if e[i]>=g[i]:

c+=0

else:

c+=-700000*(g[i]-e[i])

#AAAAAAAAAAAAAAAAAAAAAAAAAAAAAAAAAAAAAAAAAAAAAAAAAAAAAAAAAAAAAAAAAAAAAAAAAAAAAAAAAAAAAAAAAAAAAAAAAAAAAAAAAAAAAAAAAAAAAAAAAAAAAAAAAAAAAAAAAAAAAAAAAAAAAAA

return c

def T79(X):

s=X

sum1=0

sum2=0

sum3=0

sum4=0

sum5=0

sum6=0

d1=np.zeros(10)

d2=np.zeros(10)

d3=np.zeros(10)

e=[]

g=[]

h=[]

i=[]

k1=np.zeros(10)

k2=np.zeros(10)

k3=np.zeros(10)

l=[]

for i in range(150):

if(i+1)%15==0:

d3[(i+1)//15-1]=s[i]-s[i-3]-s[i-6]-s[i-9]-s[i-12]

d2[(i+1)//15-1]=s[i-1]-s[i-4]-s[i-7]-s[i-10]-s[i-13]

d1[(i+1)//15-1]=s[i-2]-s[i-5]-s[i-8]-s[i-11]-s[i-14]

for i in range(30):

if i%3==0:

e.append(d1[i//3])

elif i%3==1:

e.append(d2[i//3])

else: e.append(d3[i//3])

g=NP[0]+NP[1]*2+NP[2]*2+NP[3]+NP[4]*2+NP[5]*2

f=np.array(e)+np.array(g)

for m in range(15):

if m%3==0:

sum1+=f[m]

h.append(sum1)

elif m%3==1:

sum2+=f[m]

h.append(sum2)

else:

sum3+=f[m]

h.append(sum3)

for m in range(15,30):

if m%3==0:

sum4+=f[m]

h.append(sum4)

elif m%3==1:

sum5+=f[m]

h.append(sum5)

else:

sum6+=f[m]

h.append(sum6)

for i in range(150):

if(i+1)%15==0:

k3[(i+1)//15-1]=s[i-3]+s[i-6]+s[i-9]+s[i-12]

k2[(i+1)//15-1]=s[i-4]+s[i-7]+s[i-10]+s[i-13]

k1[(i+1)//15-1]=s[i-5]+s[i-8]+s[i-11]+s[i-14]

for i in range(30):

if i%3==0:

l.append(k1[i//3])

elif i%3==1:

l.append(k2[i//3])

else: l.append(k3[i//3])

z=0

y=0

for i in range(30):

if ((i>=0)and(i<=2))or((i>=15)and(i<=17)):

if l[i]<=g[i]:

z+=0

else:z+=-120*(l[i]-g[i])

else :

if l[i]<=g[i]+h[i-3]:

z+=0

else:z+=-120*(l[i]-g[i]-h[i-3])

o=0

for i in range(15):

if i%3==0:

o=0

elif i%3==1:

o=1

else:

o=2

if 1.1*h[i+15]+h[i]<=K0[o]:

y+=0

else:y+=-120*(1.1*h[i+15]+h[i]-K0[o])

return z+y

s=fitness1(X)-fitness2(X)-fitness34(X)-fitness5(X)-fitness67(X)-fitness89(X)-fitness10(X)+T35(X)+T46(X)+T79(X)

return s

# Initialization

def init_Population(self):

b=B[0]+B[1]*2+B[2]*2+B[3]+B[4]*2+B[5]*2

for i in range(self.pN):

for j in range(self.dim):

if j <150:

self.V[i][j] = random.uniform(-5, 5)

if j%3==0:

self.X[i][j] = round(random.randint(0, b[j//3]))

self.X[i][j+1] = round(random.randint(0, b[j//3]-self.X[i][j]))

self.X[i][j+2] =round(b[j//3]-self.X[i][j]-self.X[i][j+1])

elif j<225:

self.X[i][j]=0

self.X[i][j+75]=0

self.X[i][j+150]=0

self.V[i][j] = random.uniform(-5, 5)

self.V[i][j+75] = random.uniform(-5, 5)

self.V[i][j+150] = random.uniform(-5, 5)

else:

self.V[i][j] = random.uniform(-5, 5)

##################################################################################### ################

self.X[i][375]=0

self.X[i][376]=0

self.X[i][377]=0

self.X[i][378]=0

self.X[i][379]=0

self.X[i][380]=0

self.X[i][381]=0

self.X[i][382]=0

self.X[i][383]=0

self.pbest[i] = self.X[i]

tmp = self.function(self.X[i])

self.p_fit[i] = tmp

if tmp > self.fit:

self.fit = tmp

self.gbest = self.X[i]

#Update position

def iterator(self):

for t in range(self.max_iter):

self.w=wmin + (wmax - wmin)*(self.max_iter - t)/self.max_iter

c1s = 2.5

c1d = 0.5

c2s = 0.5

c2d = 2.5

self.c1 = (c1s - c1d)*(self.max_iter - t)/self.max_iter + c1d

self.c2 = (c2s - c2d)*(self.max_iter - t)/self.max_iter + c2d

for i in range(self.pN): # Update gbest\pbest

temp = self.function(self.X[i])

if temp > self.p_fit[i]: # pbest

self.p_fit[i] = temp

self.pbest[i] = self.X[i]

if self.p_fit[i] > self.fit: # gbest

self.gbest = self.X[i]

self.fit = self.p_fit[i]

for k in range(self.dim):

self.r1=random.random()

self.r2=random.random()

self.V[i][k] = self.w * self.V[i][k] + self.c1 * self.r1 * (self.pbest[i][k] - self.X[i][k]) + self.c2 * self.r2 * (self.gbest[k] - self.X[i][k])#Update velocity

# velocity

if k <150:

# velocity

if self.V[i][k]<-10:

self.V[i][k] = -10

if self.V[i][k]>10:

self.V[i][k] = 10

else:

if self.V[i][k]<-10:

self.V[i][k] = -10

if self.V[i][k]>10:

self.V[i][k] = 10

self.X[i] = np.round(self.X[i] + self.V[i])#Update position

self.X[i] =abs(self.X[i])

fitness.append(np.round(self.fit))

print(self.gbest//1, end=" ")

print(self.fit//1)# Output optimal value

#####################################################################################

print(self.function(self.X[1])//1)

if t==(self.max_iter-1):

for i in range(384):

QA.append(self.gbest[i])

return fitness

#Program

Fitness3=[]

QA3=[]

for i in range(3):#1

for j in range(i+1,4):#2

for k in range(j+1,5):

QA=[]

F=[]#[1,2,3,4,5]

#Distance

#[i1,i2,i3]

#Turnover times

fitness=[]

# Transportation cost

C=[]#12345

#Carrying capacity

D=[]#j20 1,2,3,4,5

#Storage capacity

#The purchased pallets

#Storage cost

#p1[i1,i2,i3]

#p2[i1,i2,i3]

#Loading and unloading cost

#p1[i1,i2,i3]

#p2[i1,i2,i3]

#CO2 g/km

h=[]

#Idle cost

o=[]

#Rental fee

p=[]

F.append(FA[i])

C.append(CA[i])

D.append(DA[i])

h.append(hA[i])

o.append(oA[i])

p.append(pA[i])

F.append(FA[j])

C.append(CA[j])

D.append(DA[j])

h.append(hA[j])

o.append(oA[j])

p.append(pA[j])

F.append(FA[k])

C.append(CA[k])

D.append(DA[k])

h.append(hA[k])

o.append(oA[k])

p.append(pA[k])

my_pso = PSO3(pN=80, dim=384, max_iter=2000)#

my_pso.init_Population()

fitness = my_pso.iterator()

Fitness3.append(fitness)

QA3.append(QA)

class PSO4():

def __init__(self, pN, dim, max_iter):

self.w = 2

self.c1 = 2

self.c2 = 2

self.r1 = 2

self.r2 = 2

self.pN = pN # Number of particles

self.dim = dim # Search dimension

self.max_iter = max_iter # Number of iterations

self.X = np.zeros((self.pN, self.dim)) # Position and velocity

self.V = np.zeros((self.pN, self.dim))

self.pbest = np.zeros((self.pN, self.dim)) # Update pbest and gbest

self.gbest = np.zeros((1, self.dim))

self.p_fit = np.zeros(self.pN) # pbest

self.fit = -9999999999999999999999999

# Objective function

def function(self, X):#X=[p1 t1 j1 i1,p1 t1 j1 i2,p1 t1 j1 i3,p1 t1 j2 i1...p1 t2 j1 i1...p2 t1 j1 i1]

def fitness1(X):

ss=np.array(X)

s=0

for i in range(150):

if(i+3)%15==0:

continue

elif(i+2)%15==0:

continue

elif (i+1)%15==0:

continue

else:

if i<75:

s+=ss[i]*72

else: s+=ss[i]*108

return s

def fitness2(X):

s=0

for i in range(12):

if i<3:

s+=X[i+450]*F[i//3]

elif i<6:

s+=X[i+450]*F[i//3]

elif i<9:

s+=X[i+450]*F[i//3]

elif i<12:

s+=X[i+450]*F[i//3]

else:

s+=X[i+450]*F[i//3]

return s

def fitness34(X):

s=X

l1=[]

A1=[]

sum=0

for i in range(5):

l1+=l[i]

A1+=A[i]

e=l1*20

f=A1*20

for i in range(300):

sum+=r*e[i]*s[i+150]*f[i]*C[i//75]

return sum

def fitness5(X):

s=X

sum1=0

sum2=0

sum3=0

sum4=0

sum5=0

sum6=0

d1=np.zeros(10)

d2=np.zeros(10)

d3=np.zeros(10)

e=[]

g=[]

h=[]

i=[]

for i in range(150):

if(i+1)%15==0:

d3[(i+1)//15-1]=s[i]-s[i-3]-s[i-6]-s[i-9]-s[i-12]#3

d2[(i+1)//15-1]=s[i-1]-s[i-4]-s[i-7]-s[i-10]-s[i-13]#2

d1[(i+1)//15-1]=s[i-2]-s[i-5]-s[i-8]-s[i-11]-s[i-14]#1

for i in range(30):

if i%3==0:

e.append(d1[i//3])

elif i%3==1:

e.append(d2[i//3])

else: e.append(d3[i//3])#e xo-xi[p1 t1 i1, p1 t1 i2]

g=NP[0]+NP[1]*2+NP[2]*2+NP[3]+NP[4]*2+NP[5]*2#the purchase pallets

f=np.array(e)+np.array(g)# +xo-xi

for m in range(15):

if m%3==0:

sum1+=f[m]

h.append(sum1)

elif m%3==1:

sum2+=f[m]

h.append(sum2)

else:

sum3+=f[m]

h.append(sum3)

for m in range(15,30):

if m%3==0:

sum4+=f[m]

h.append(sum4)

elif m%3==1:

sum5+=f[m]

h.append(sum5)

else:

sum6+=f[m]

h.append(sum6)

sum=0

i=pk1*5+pk2*5

for m in range(30):

sum+=np.array(h[m])*np.array(i[m])

return sum

def fitness67(X):

pl=pl1*25+pl2*25

s=X

sum=0

for i in range(150):

sum+=s[i]*pl[i]

return sum

def fitness89(X):

s=np.array(X)

sum=0

d=np.zeros(60)#vijkt

e=np.zeros(60)#vik

f=np.zeros(60)#xianzhi

g=np.zeros(60)#zu

a=0

b=0

c=0

for i in range(300):

if i%15==0:

d[(i//15)*3]=s[150+i]+s[153+i]+s[156+i]+s[159+i]

d[(i//15)*3+1]=s[151+i]+s[154+i]+s[157+i]+s[160+i]

d[(i//15)*3+2]=s[152+i]+s[155+i]+s[158+i]+s[161+i]

for i in range(4):

e[15*i+0]=s[i*3+450]

e[15*i+1]=s[i*3+451]

e[15*i+2]=s[i*3+452]

e[15*i+3]=s[i*3+450]

e[15*i+4]=s[i*3+451]

e[15*i+5]=s[i*3+452]

e[15*i+6]=s[i*3+450]

e[15*i+7]=s[i*3+451]

e[15*i+8]=s[i*3+452]

e[15*i+9]=s[i*3+450]

e[15*i+10]=s[i*3+451]

e[15*i+11]=s[i*3+452]

e[15*i+12]=s[i*3+450]

e[15*i+13]=s[i*3+451]

e[15*i+14]=s[i*3+452]

for i in range(60):

if i <15:

f[i]=o[0]

elif i<30:

f[i]=o[1]

elif i<45:

f[i]=o[2]

elif i<60:

f[i]=o[3]

else:

f[i]=o[4]

for i in range(60):

if i <15:

g[i]=p[0]

elif i<30:

g[i]=p[1]

elif i<45:

g[i]=p[2]

elif i<60:

g[i]=p[3]

else:

g[i]=p[4]

for i in range(60):

if e[i]>d[i]:

sum+=f[i]*(e[i]-d[i])

else:

sum+=g[i]*(d[i]-e[i])

return sum

def fitness10(X):

s=X

l1=[]

A1=[]

sum=0

for i in range(5):

l1+=l[i]

A1+=A[i]

e=l1*20

f=A1*20

for i in range(300):

sum+=r*e[i]*s[i+150]*f[i]*gg*h[i//75]

return sum

def T35(X):

c=0

s=[]

b=B[0]+B[1]*2+B[2]*2+B[3]+B[4]*2+B[5]*2

for i in range(0,150):

if i%3==0:

s.append(X[i]+X[i+1]+X[i+2])

for i in range(0,50):

if (i+1)%5==0:

if s[i]>=b[i]:

c+=0

else:c+=-120*(b[i]-s[i])

else:

if s[i]<=b[i]:

c+=0

else:c+=-120*(s[i]-b[i])

return c

def T46(X):

s=X

A1=[]

g=np.zeros(75)

for i in range(5):

A1+=A[i]

f=A1*20

c=0

d=np.zeros(300)

e=np.zeros(75)

for i in range(75):

d[i]=D[0]

for i in range(75,150):

d[i]=D[1]

for i in range(150,225):

d[i]=D[2]

for i in range(225,300):

d[i]=D[3]

for i in range(75):

e[i]=r*(d[i]*f[i]*s[i+150]+d[i+75]*f[i+75]*s[i+225]+d[i+150]*f[i+150]*s[i+300]+d[i+225]*f[i+225]*s[i+375])

for i in range(75):

g[i]=s[i]+s[i+75]*1.1

for i in range(75):

if e[i]>=g[i]:

c+=0

else:

c+=-700000*(g[i]-e[i])

#AAAAAAAAAAAAAAAAAAAAAAAAAAAAAAAAAAAAAAAAAAAAAAAAAAAAAAAAAAAAAAAAAAAAAAAAAAAAAAAAAAAAAAAAAAAAAAAAAAAAAAAAAAAAAAAAAAAAAAAAAAAAAAAAAAAAAAAAAAAAAAAAAAAAAAA

return c

def T79(X):

s=X

sum1=0

sum2=0

sum3=0

sum4=0

sum5=0

sum6=0

d1=np.zeros(10)

d2=np.zeros(10)

d3=np.zeros(10)

e=[]

g=[]

h=[]

i=[]

k1=np.zeros(10)

k2=np.zeros(10)

k3=np.zeros(10)

l=[]

for i in range(150):

if(i+1)%15==0:

d3[(i+1)//15-1]=s[i]-s[i-3]-s[i-6]-s[i-9]-s[i-12]

d2[(i+1)//15-1]=s[i-1]-s[i-4]-s[i-7]-s[i-10]-s[i-13]

d1[(i+1)//15-1]=s[i-2]-s[i-5]-s[i-8]-s[i-11]-s[i-14]

for i in range(30):

if i%3==0:

e.append(d1[i//3])

elif i%3==1:

e.append(d2[i//3])

else: e.append(d3[i//3])

g=NP[0]+NP[1]*2+NP[2]*2+NP[3]+NP[4]*2+NP[5]*2

f=np.array(e)+np.array(g)

for m in range(15):

if m%3==0:

sum1+=f[m]

h.append(sum1)

elif m%3==1:

sum2+=f[m]

h.append(sum2)

else:

sum3+=f[m]

h.append(sum3)

for m in range(15,30):

if m%3==0:

sum4+=f[m]

h.append(sum4)

elif m%3==1:

sum5+=f[m]

h.append(sum5)

else:

sum6+=f[m]

h.append(sum6)

for i in range(150):

if(i+1)%15==0:

k3[(i+1)//15-1]=s[i-3]+s[i-6]+s[i-9]+s[i-12]

k2[(i+1)//15-1]=s[i-4]+s[i-7]+s[i-10]+s[i-13]

k1[(i+1)//15-1]=s[i-5]+s[i-8]+s[i-11]+s[i-14]

for i in range(30):

if i%3==0:

l.append(k1[i//3])

elif i%3==1:

l.append(k2[i//3])

else: l.append(k3[i//3])

z=0

y=0

for i in range(30):

if ((i>=0)and(i<=2))or((i>=15)and(i<=17)):

if l[i]<=g[i]:

z+=0

else:z+=-120*(l[i]-g[i])

else :

if l[i]<=g[i]+h[i-3]:

z+=0

else:z+=-120*(l[i]-g[i]-h[i-3])

o=0

for i in range(15):

if i%3==0:

o=0

elif i%3==1:

o=1

else:

o=2

if 1.1*h[i+15]+h[i]<=K0[o]:

y+=0

else:y+=-120*(1.1*h[i+15]+h[i]-K0[o])

return z+y

s=fitness1(X)-fitness2(X)-fitness34(X)-fitness5(X)-fitness67(X)-fitness89(X)-fitness10(X)+T35(X)+T46(X)+T79(X)

return s

# Initialization

def init_Population(self):

b=B[0]+B[1]*2+B[2]*2+B[3]+B[4]*2+B[5]*2

for i in range(self.pN):

y11=0

y21=0

y31=0

y12=0

y22=0

y32=0

y13=0

y23=0

y33=0

y14=0

y24=0

y34=0

for j in range(self.dim):

if j <150:

self.V[i][j] = random.uniform(-5, 5)

if j%3==0:

self.X[i][j] = round(random.randint(0, b[j//3]))

self.X[i][j+1] = round(random.randint(0, b[j//3]-self.X[i][j]))

self.X[i][j+2] =round(b[j//3]-self.X[i][j]-self.X[i][j+1])

elif j<225:

self.X[i][j]=0

self.X[i][j+75]=0

self.X[i][j+150]=0

self.X[i][j+225]=0

self.V[i][j] = random.uniform(-5, 5)

self.V[i][j+75] = random.uniform(-5, 5)

self.V[i][j+150] = random.uniform(-5, 5)

self.V[i][j+225] = random.uniform(-5, 5)

elif j >=450:

self.V[i][j] = random.uniform(-5, 5)

##################################################################################### ################

for t in range(75):

if t%3==0:

y11+=self.X[i][t+150]

y21+=self.X[i][t+151]

y31+=self.X[i][t+152]

y12+=self.X[i][t+225]

y22+=self.X[i][t+226]

y32+=self.X[i][t+227]

y13+=self.X[i][t+300]

y23+=self.X[i][t+301]

y33+=self.X[i][t+302]

y14+=self.X[i][t+375]

y24+=self.X[i][t+376]

y34+=self.X[i][t+377]

self.X[i][450]=0

self.X[i][451]=0

self.X[i][452]=0

self.X[i][453]=0

self.X[i][454]=0

self.X[i][455]=0

self.X[i][456]=0

self.X[i][457]=0

self.X[i][458]=0

self.X[i][459]=0

self.X[i][460]=0

self.X[i][461]=0

self.pbest[i] = self.X[i]

tmp = self.function(self.X[i])

self.p_fit[i] = tmp

if tmp > self.fit:

self.fit = tmp

self.gbest = self.X[i]

#Update position

def iterator(self):

for t in range(self.max_iter):

self.w=wmin + (wmax - wmin)*(self.max_iter - t)/self.max_iter

c1s = 2.5

c1d = 0.5

c2s = 0.5

c2d = 2.5

self.c1 = (c1s - c1d)*(self.max_iter - t)/self.max_iter + c1d

self.c2 = (c2s - c2d)*(self.max_iter - t)/self.max_iter + c2d

for i in range(self.pN): # Update gbest\pbest

temp = self.function(self.X[i])

if temp > self.p_fit[i]: # Update pbest

self.p_fit[i] = temp

self.pbest[i] = self.X[i]

if self.p_fit[i] > self.fit: # Update gbest

self.gbest = self.X[i]

self.fit = self.p_fit[i]

for k in range(self.dim):

self.r1=random.random()

self.r2=random.random()

self.V[i][k] = self.w * self.V[i][k] + self.c1 * self.r1 * (self.pbest[i][k] - self.X[i][k]) + self.c2 * self.r2 * (self.gbest[k] - self.X[i][k])#Update velocity

# velocity

if k <150:

# velocity

if self.V[i][k]<-10:

self.V[i][k] = -10

if self.V[i][k]>10:

self.V[i][k] = 10

else:

if self.V[i][k]<-10:

self.V[i][k] = -10

if self.V[i][k]>10:

self.V[i][k] = 10

self.X[i] = np.round(self.X[i] + self.V[i])#Update position

self.X[i] =abs(self.X[i])

fitness.append(np.round(self.fit))

print(self.gbest//1, end=" ")

print(self.fit//1)# Output optimal value

#####################################################################################

print(self.function(self.X[1])//1)

if t==(self.max_iter-1):

for i in range(462):

QA.append(self.gbest[i])

return fitness

#Program

Fitness4=[]

QA4=[]

for i in range(2):#1

for j in range(i+1,3):#2

for k in range(j+1,4):

for ii in range(k+1,5):#3

F=[]#[1,2,3,4,5]

#Distance

#[i1,i2,i3]

QA=[]

#Turnover times

fitness=[]

#Transportation cost

C=[]#12345

#Carrying capacity

D=[]#j20 1,2,3,4,5

#Storage capacity

#The purchased pallets

#Storage cost

#p1[i1,i2,i3]

#p2[i1,i2,i3]

#Loading and unloading cost

#p1[i1,i2,i3]

#p2[i1,i2,i3]

#CO2 g/km

h=[]

#Idle cost

o=[]

#Rental fee

p=[]

F.append(FA[i])

C.append(CA[i])

D.append(DA[i])

h.append(hA[i])

o.append(oA[i])

p.append(pA[i])

F.append(FA[j])

C.append(CA[j])

D.append(DA[j])

h.append(hA[j])

o.append(oA[j])

p.append(pA[j])

F.append(FA[k])

C.append(CA[k])

D.append(DA[k])

h.append(hA[k])

o.append(oA[k])

p.append(pA[k])

F.append(FA[ii])

C.append(CA[ii])

D.append(DA[ii])

h.append(hA[ii])

o.append(oA[ii])

p.append(pA[ii])

my_pso = PSO4(pN=80, dim=462, max_iter=2000)#

my_pso.init_Population()

fitness = my_pso.iterator()

Fitness4.append(fitness)

QA4.append(QA)

#PSO

class PSO5():

def __init__(self, pN, dim, max_iter):

self.w = 2

self.c1 = 2

self.c2 = 2

self.r1 = 2

self.r2 = 2

self.pN = pN # Number of particles

self.dim = dim # Search dimension

self.max_iter = max_iter # Number of iterations

self.X = np.zeros((self.pN, self.dim)) # Position and velocity

self.V = np.zeros((self.pN, self.dim))

self.pbest = np.zeros((self.pN, self.dim)) # pbest and gbest

self.gbest = np.zeros((1, self.dim))

self.p_fit = np.zeros(self.pN) # pbest

self.fit = -9999999999999999999999999

# Objective function

def function(self, X):#X=[p1 t1 j1 i1,p1 t1 j1 i2,p1 t1 j1 i3,p1 t1 j2 i1...p1 t2 j1 i1...p2 t1 j1 i1]

def fitness1(X):

ss=np.array(X)

s=0

for i in range(150):

if(i+3)%15==0:

continue

elif(i+2)%15==0:

continue

elif (i+1)%15==0:

continue

else:

if i<75:

s+=ss[i]*72

else: s+=ss[i]*108

return s

def fitness2(X):

s=0

for i in range(15):

if i<3:

s+=X[i+525]*F[i//3]

elif i<6:

s+=X[i+525]*F[i//3]

elif i<9:

s+=X[i+525]*F[i//3]

elif i<12:

s+=X[i+525]*F[i//3]

else:

s+=X[i+525]*F[i//3]

return s

def fitness34(X):

s=X

l1=[]

A1=[]

sum=0

for i in range(5):

l1+=l[i]

A1+=A[i]

e=l1*25

f=A1*25

for i in range(375):

sum+=r*e[i]*s[i+150]*f[i]*C[i//75]

return sum

def fitness5(X):

s=X

sum1=0

sum2=0

sum3=0

sum4=0

sum5=0

sum6=0

d1=np.zeros(10)

d2=np.zeros(10)

d3=np.zeros(10)

e=[]

g=[]

h=[]

i=[]

for i in range(150):

if(i+1)%15==0:

d3[(i+1)//15-1]=s[i]-s[i-3]-s[i-6]-s[i-9]-s[i-12]#3

d2[(i+1)//15-1]=s[i-1]-s[i-4]-s[i-7]-s[i-10]-s[i-13]#2

d1[(i+1)//15-1]=s[i-2]-s[i-5]-s[i-8]-s[i-11]-s[i-14]#1

for i in range(30):

if i%3==0:

e.append(d1[i//3])

elif i%3==1:

e.append(d2[i//3])

else: e.append(d3[i//3])#e xo-xi[p1 t1 i1, p1 t1 i2]

g=NP[0]+NP[1]*2+NP[2]*2+NP[3]+NP[4]*2+NP[5]*2#The purchase pallets

f=np.array(e)+np.array(g)# +xo-xi

for m in range(15):

if m%3==0:

sum1+=f[m]

h.append(sum1)

elif m%3==1:

sum2+=f[m]

h.append(sum2)

else:

sum3+=f[m]

h.append(sum3)

for m in range(15,30):

if m%3==0:

sum4+=f[m]

h.append(sum4)

elif m%3==1:

sum5+=f[m]

h.append(sum5)

else:

sum6+=f[m]

h.append(sum6)

sum=0

i=pk1*5+pk2*5

for m in range(30):

sum+=np.array(h[m])*np.array(i[m])

return sum

def fitness67(X):

pl=pl1*25+pl2*25

s=X

sum=0

for i in range(150):

sum+=s[i]*pl[i]

return sum

def fitness89(X):

s=np.array(X)

sum=0

d=np.zeros(75)#vijkt

e=np.zeros(75)#vik

f=np.zeros(75)#xianzhi

g=np.zeros(75)#zu

a=0

b=0

c=0

for i in range(375):

if i%15==0:

d[(i//15)*3]=s[150+i]+s[153+i]+s[156+i]+s[159+i]

d[(i//15)*3+1]=s[151+i]+s[154+i]+s[157+i]+s[160+i]

d[(i//15)*3+2]=s[152+i]+s[155+i]+s[158+i]+s[161+i]

for i in range(5):

e[15*i+0]=s[i*3+525]

e[15*i+1]=s[i*3+526]

e[15*i+2]=s[i*3+527]

e[15*i+3]=s[i*3+525]

e[15*i+4]=s[i*3+526]

e[15*i+5]=s[i*3+527]

e[15*i+6]=s[i*3+525]

e[15*i+7]=s[i*3+526]

e[15*i+8]=s[i*3+527]

e[15*i+9]=s[i*3+525]

e[15*i+10]=s[i*3+526]

e[15*i+11]=s[i*3+527]

e[15*i+12]=s[i*3+525]

e[15*i+13]=s[i*3+526]

e[15*i+14]=s[i*3+527]

for i in range(75):

if i <15:

f[i]=o[0]

elif i<30:

f[i]=o[1]

elif i<45:

f[i]=o[2]

elif i<60:

f[i]=o[3]

else:

f[i]=o[4]

for i in range(75):

if i <15:

g[i]=p[0]

elif i<30:

g[i]=p[1]

elif i<45:

g[i]=p[2]

elif i<60:

g[i]=p[3]

else:

g[i]=p[4]

for i in range(75):

if e[i]>d[i]:

sum+=f[i]*(e[i]-d[i])

else:

sum+=g[i]*(d[i]-e[i])

return sum

def fitness10(X):

s=X

l1=[]

A1=[]

sum=0

for i in range(5):

l1+=l[i]

A1+=A[i]

e=l1*25

f=A1*25

for i in range(375):

sum+=r*e[i]*s[i+150]*f[i]*gg*h[i//75]

return sum

def T35(X):

c=0

s=[]

b=B[0]+B[1]*2+B[2]*2+B[3]+B[4]*2+B[5]*2

for i in range(0,150):

if i%3==0:

s.append(X[i]+X[i+1]+X[i+2])

for i in range(0,50):

if (i+1)%5==0:

if s[i]>=b[i]:

c+=0

else:c+=-120*(b[i]-s[i])

else:

if s[i]<=b[i]:

c+=0

else:c+=-120*(s[i]-b[i])

return c

def T46(X):

s=X

A1=[]

g=np.zeros(75)

for i in range(5):

A1+=A[i]

f=A1*25

c=0

d=np.zeros(375)

e=np.zeros(75)

for i in range(75):

d[i]=D[0]

for i in range(75,150):

d[i]=D[1]

for i in range(150,225):

d[i]=D[2]

for i in range(225,300):

d[i]=D[3]

for i in range(300,375):

d[i]=D[4]

for i in range(75):

e[i]=r*(d[i]*f[i]*s[i+150]+d[i+75]*f[i+75]*s[i+225]+d[i+150]*f[i+150]*s[i+300]+d[i+225]*f[i+225]*s[i+375]+d[i+300]*f[i+300]*s[i+450])

for i in range(75):

g[i]=s[i]+s[i+75]*1.1

for i in range(75):

if e[i]>=g[i]:

c+=0

else:

c+=-700000*(g[i]-e[i])

#AAAAAAAAAAAAAAAAAAAAAAAAAAAAAAAAAAAAAAAAAAAAAAAAAAAAAAAAAAAAAAAAAAAAAAAAAAAAAAAAAAAAAAAAAAAAAAAAAAAAAAAAAAAAAAAAAAAAAAAAAAAAAAAAAAAAAAAAAAAAAAAAAAAAAAA

return c

def T79(X):

s=X

sum1=0

sum2=0

sum3=0

sum4=0

sum5=0

sum6=0

d1=np.zeros(10)

d2=np.zeros(10)

d3=np.zeros(10)

e=[]

g=[]

h=[]

i=[]

k1=np.zeros(10)

k2=np.zeros(10)

k3=np.zeros(10)

l=[]

for i in range(150):

if(i+1)%15==0:

d3[(i+1)//15-1]=s[i]-s[i-3]-s[i-6]-s[i-9]-s[i-12]

d2[(i+1)//15-1]=s[i-1]-s[i-4]-s[i-7]-s[i-10]-s[i-13]

d1[(i+1)//15-1]=s[i-2]-s[i-5]-s[i-8]-s[i-11]-s[i-14]

for i in range(30):

if i%3==0:

e.append(d1[i//3])

elif i%3==1:

e.append(d2[i//3])

else: e.append(d3[i//3])

g=NP[0]+NP[1]*2+NP[2]*2+NP[3]+NP[4]*2+NP[5]*2

f=np.array(e)+np.array(g)

for m in range(15):

if m%3==0:

sum1+=f[m]

h.append(sum1)

elif m%3==1:

sum2+=f[m]

h.append(sum2)

else:

sum3+=f[m]

h.append(sum3)

for m in range(15,30):

if m%3==0:

sum4+=f[m]

h.append(sum4)

elif m%3==1:

sum5+=f[m]

h.append(sum5)

else:

sum6+=f[m]

h.append(sum6)

for i in range(150):

if(i+1)%15==0:

k3[(i+1)//15-1]=s[i-3]+s[i-6]+s[i-9]+s[i-12]

k2[(i+1)//15-1]=s[i-4]+s[i-7]+s[i-10]+s[i-13]

k1[(i+1)//15-1]=s[i-5]+s[i-8]+s[i-11]+s[i-14]

for i in range(30):

if i%3==0:

l.append(k1[i//3])

elif i%3==1:

l.append(k2[i//3])

else: l.append(k3[i//3])

z=0

y=0

for i in range(30):

if ((i>=0)and(i<=2))or((i>=15)and(i<=17)):

if l[i]<=g[i]:

z+=0

else:z+=-120*(l[i]-g[i])

else :

if l[i]<=g[i]+h[i-3]:

z+=0

else:z+=-120*(l[i]-g[i]-h[i-3])

o=0

for i in range(15):

if i%3==0:

o=0

elif i%3==1:

o=1

else:

o=2

if 1.1*h[i+15]+h[i]<=K0[o]:

y+=0

else:y+=-120*(1.1*h[i+15]+h[i]-K0[o])

return z+y

s=fitness1(X)-fitness2(X)-fitness34(X)-fitness5(X)-fitness67(X)-fitness89(X)-fitness10(X)+T35(X)+T46(X)+T79(X)

return s

#Initialization

def init_Population(self):

b=B[0]+B[1]*2+B[2]*2+B[3]+B[4]*2+B[5]*2

for i in range(self.pN):

for j in range(self.dim):

if j <150:

self.V[i][j] = random.uniform(-5, 5)

if j%3==0:

self.X[i][j] = round(random.randint(0, b[j//3]))

self.X[i][j+1] = round(random.randint(0, b[j//3]-self.X[i][j]))

self.X[i][j+2] =round(b[j//3]-self.X[i][j]-self.X[i][j+1])

elif j<225:

self.X[i][j]=0

self.X[i][j+75]=0

self.X[i][j+150]=0

self.X[i][j+225]=0

self.X[i][j+300]=0

self.V[i][j] = random.uniform(-5, 5)

self.V[i][j+75] = random.uniform(-5, 5)

self.V[i][j+150] = random.uniform(-5, 5)

self.V[i][j+225] = random.uniform(-5, 5)

self.V[i][j+300] = random.uniform(-5, 5)

else:

self.V[i][j] = random.uniform(-5, 5)

##################################################################################### ################

self.X[i][525]=0

self.X[i][526]=0

self.X[i][527]=0

self.X[i][528]=0

self.X[i][529]=0

self.X[i][530]=0

self.X[i][531]=0

self.X[i][532]=0

self.X[i][533]=0

self.X[i][534]=0

self.X[i][535]=0

self.X[i][536]=0

self.X[i][537]=0

self.X[i][538]=0

self.X[i][539]=0

self.pbest[i] = self.X[i]

tmp = self.function(self.X[i])

self.p_fit[i] = tmp

if tmp > self.fit:

self.fit = tmp

self.gbest = self.X[i]

#Update position

def iterator(self):

for t in range(self.max_iter):

self.w=wmin + (wmax - wmin)*(self.max_iter - t)/self.max_iter

c1s = 2.5

c1d = 0.5

c2s = 0.5

c2d = 2.5

self.c1 = (c1s - c1d)*(self.max_iter - t)/self.max_iter + c1d

self.c2 = (c2s - c2d)*(self.max_iter - t)/self.max_iter + c2d

for i in range(self.pN): # Update gbest\pbest

temp = self.function(self.X[i])

if temp > self.p_fit[i]: # Update pbest

self.p_fit[i] = temp

self.pbest[i] = self.X[i]

if self.p_fit[i] > self.fit: # Update gbest

self.gbest = self.X[i]

self.fit = self.p_fit[i]

for k in range(self.dim):

self.r1=random.random()

self.r2=random.random()

self.V[i][k] = self.w * self.V[i][k] + self.c1 * self.r1 * (self.pbest[i][k] - self.X[i][k]) + self.c2 * self.r2 * (self.gbest[k] - self.X[i][k])#Update velocity

# velocity

if k <150:

# velocity

if self.V[i][k]<-10:

self.V[i][k] = -10

if self.V[i][k]>10:

self.V[i][k] = 10

else:

if self.V[i][k]<-10:

self.V[i][k] = -10

if self.V[i][k]>10:

self.V[i][k] = 10

self.X[i] = np.round(self.X[i] + self.V[i])#Update position

self.X[i] =abs(self.X[i])

fitness.append(np.round(self.fit))

print(self.gbest//1, end=" ")

print(self.fit//1)# Output optimal value

#####################################################################################

print(self.function(self.X[1])//1)

if t==(self.max_iter-1):

for i in range(540):

QA.append(self.gbest[i])

return fitness

#Program

Fitness5=[]

QA5=[]

for i in range(1):#1

for j in range(i+1,2):#2

for k in range(j+1,3):

for ii in range(k+1,4):

for iii in range(ii+1,5):#3

F=[]#[1,2,3,4,5]

#Distance

#[i1,i2,i3]

QA=[]

#Turnover times

fitness=[]

#Transportation cost

C=[]#12345

#Carrying capacity

D=[]#j20 1,2,3,4,5

#Storage cost

#p1[i1,i2,i3]

#p2[i1,i2,i3]

#Loading and unloading cost

#p1[i1,i2,i3]

#p2[i1,i2,i3]

#CO2 g/km

h=[]

#Idle cost

o=[]

#Rental fee

p=[]

F.append(FA[i])

C.append(CA[i])

D.append(DA[i])

h.append(hA[i])

o.append(oA[i])

p.append(pA[i])

F.append(FA[j])

C.append(CA[j])

D.append(DA[j])

h.append(hA[j])

o.append(oA[j])

p.append(pA[j])

F.append(FA[k])

C.append(CA[k])

D.append(DA[k])

h.append(hA[k])

o.append(oA[k])

p.append(pA[k])

F.append(FA[ii])

C.append(CA[ii])

D.append(DA[ii])

h.append(hA[ii])

o.append(oA[ii])

p.append(pA[ii])

F.append(FA[iii])

C.append(CA[iii])

D.append(DA[iii])

h.append(hA[iii])

o.append(oA[iii])

p.append(pA[iii])

my_pso = PSO5(pN=80, dim=540, max_iter=2000)#

my_pso.init_Population()

fitness = my_pso.iterator()

Fitness5.append(fitness)

QA5.append(QA)

FI=[]

S=[]

for i in range(2000):

SS=[Fitness1[0][i],Fitness1[1][i],Fitness1[2][i],Fitness1[3][i],Fitness1[4][i],Fitness2[0][i],Fitness2[1][i],Fitness2[2][i],Fitness2[3][i],Fitness2[4][i],Fitness2[5][i],Fitness2[6][i],Fitness2[7][i],Fitness2[8][i],Fitness2[9][i],Fitness3[0][i],Fitness3[1][i],Fitness3[2][i],Fitness3[3][i],Fitness3[4][i],Fitness3[5][i],Fitness3[6][i],Fitness3[7][i],Fitness3[8][i],Fitness3[9][i],Fitness4[0][i],Fitness4[1][i],Fitness4[2][i],Fitness4[3][i],Fitness4[4][i],Fitness5[0][i]]

FI.append(max(SS))

S.append(SS.index(max(SS)))

print(FI,S)

#0 1

#1 2

#2 3

#3 4

#4 5

#6-1 12

#7-1 13

#8-1 14

#9-1 15

#10-1 23

#11-1 24

#12-1 25

#13-1 34

#14-1 35

#15-1 45

#16-1 123

#17-1 124

#18-1 125

#19-1 134

#20-1 135

#21-1 145

#22-1 234

#23-1 235

#24-1 245

#25-1 345

#26-1 1234

#27-1 1235

#28-1 1245

#29-1 1345

#30-1 2345

#31-1 12345

end = time.time()

print("Time used:",end-start)

#excel

def write_excel():

workbook = xlsxwriter.Workbook('chat.xlsx')# excel

worksheet = workbook.add_worksheet(u'sheet1')# sheet

worksheet.set_column('A:A',20)

bold= workbook.add_format({'bold':True})

for i in range(2000):

worksheet.write(2,i,i)

worksheet.write(3,i,FI[i])

worksheet.write(4,i,S[i])

worksheet = workbook.add_worksheet(u'sheet1K')

worksheet.set_column('A:A',20)

bold= workbook.add_format({'bold':True})

for i in range(228):

worksheet.write(2,i,i)

worksheet.write(3,i,QA1[0][i])

worksheet.write(4,i,QA1[1][i])

worksheet.write(5,i,QA1[2][i])

worksheet.write(6,i,QA1[3][i])

worksheet.write(7,i,QA1[4][i])

worksheet = workbook.add_worksheet(u'sheet2K')

worksheet.set_column('A:A',20)

bold= workbook.add_format({'bold':True})

for i in range(306):

worksheet.write(2,i,i)

worksheet.write(3,i,QA2[0][i])

worksheet.write(4,i,QA2[1][i])

worksheet.write(5,i,QA2[2][i])

worksheet.write(6,i,QA2[3][i])

worksheet.write(7,i,QA2[4][i])

worksheet.write(8,i,QA2[5][i])

worksheet.write(9,i,QA2[6][i])

worksheet.write(10,i,QA2[7][i])

worksheet.write(11,i,QA2[8][i])

worksheet.write(12,i,QA2[9][i])

worksheet = workbook.add_worksheet(u'sheet3K')

worksheet.set_column('A:A',20)

bold= workbook.add_format({'bold':True})

for i in range(384):

worksheet.write(2,i,i)

worksheet.write(3,i,QA3[0][i])

worksheet.write(4,i,QA3[1][i])

worksheet.write(5,i,QA3[2][i])

worksheet.write(6,i,QA3[3][i])

worksheet.write(7,i,QA3[4][i])

worksheet.write(8,i,QA3[5][i])

worksheet.write(9,i,QA3[6][i])

worksheet.write(10,i,QA3[7][i])

worksheet.write(11,i,QA3[8][i])

worksheet.write(12,i,QA3[9][i])

worksheet = workbook.add_worksheet(u'sheet4K')

worksheet.set_column('A:A',20)

bold= workbook.add_format({'bold':True})

for i in range(462):

worksheet.write(2,i,i)

worksheet.write(3,i,QA4[0][i])

worksheet.write(4,i,QA4[1][i])

worksheet.write(5,i,QA4[2][i])

worksheet.write(6,i,QA4[3][i])

worksheet.write(7,i,QA4[4][i])

worksheet = workbook.add_worksheet(u'sheet5K')

worksheet.set_column('A:A',20)

bold= workbook.add_format({'bold':True})

for i in range(540):

worksheet.write(2,i,i)

worksheet.write(3,i,QA5[0][i])

workbook.close()

if __name__ == '__main__':

# Excel

write_excel();

print ('Success')
