# Supplementary material for: An optimization model for fleet sizing and empty pallet allocation considering CO2 emissions
Source: PLoS One. 2020 Feb 21;15(2):e0229544. doi: 10.1371/journal.pone.0229544 (PMC7034922; doi:10.1371/journal.pone.0229544)
Supplement: S2 Code — (DOCX) [file pone.0229544.s002.docx]

**S2 Code. Lingo**

MODEL:

sets:

supplier/1..3/:capacity,

maxst,

stockc,stocko,stock,

k,kk,kkk,kkkk,kkkkk,

rk,rkk,rkkk,rkkkk,rkkkkk;

qcustomer/1..4/:demand;

fcustomer/1/:fdemand;

linkssq(supplier,qcustomer):dis,time,vol,vk,vkk,vkkk,vkkkk,vkkkkk;

linksfs(fcustomer,supplier):fdis,ftime,fvol,fvk,fvkk,fvkkk,fvkkkk,fvkkkkk;

endsets

data:

!about supplier;

capacity=4000 4000 4000;

stockc=0.1 0.2 0.2;

maxst=60000 80000 80000;

stocko=0 0 0;

!about demand area;

demand=2000 1600 1500 1200;

!about supply area;

fdemand=6300;

!abouti,j;

dis=50 60 40 65

55 55 40 60

20 30 30 40;

time=10 10 15 10

10 10 15 10

15 15 15 15;

!about f,i;

fdis=70 75 50;

ftime=10 10 10;

enddata

!Set(15), objective function;

max=@sum(linkssq(i,j):72*vol(i,j))-

@sum(supplier(i):k(i)*400000+kk(i)*300000+kkk(i)*200000+kkkk(i)*90000+kkkkk(i)*90000)-

@sum(linkssq(i,j):(0.75*time(i,j)*dis(i,j)*vk(i,j)+0.6*time(i,j)*dis(i,j)*vkk(i,j)+0.55*time(i,j)*dis(i,j)*vkkk(i,j)+0.4*time(i,j)*dis(i,j)*vkkkk(i,j)+0.4*time(i,j)*dis(i,j)*vkkkkk(i,j)))-

@sum(linksfs(f,i):(0.75*ftime(f,i)*fdis(f,i)*fvk(f,i)+0.6*ftime(f,i)*fdis(f,i)*fvkk(f,i)+0.55*ftime(f,i)*fdis(f,i)*fvkkk(f,i)+0.4*ftime(f,i)*fdis(f,i)*fvkkkk(f,i)+0.4*ftime(f,i)*fdis(f,i)*fvkkkkk(f,i)))-

@sum(supplier(i):stockc(i)*stock(i))-

@sum(linkssq(i,j):0.12*vol(i,j))-

@sum(linksfs(f,i):0.12*fvol(f,i))-

100*@if((@sum(supplier(i):k(i)+rk(i)-@sum(qcustomer(j):vk(i,j)))) #le#0, 0, @sum(supplier(i):k(i)+rk(i)-@sum(qcustomer(j):vk(i,j))) )-

90*@if((@sum(supplier(i):kk(i)+rkk(i)-@sum(qcustomer(j):vkk(i,j))) )#le#0, 0, @sum(supplier(i):kk(i)+rkk(i)-@sum(qcustomer(j):vkk(i,j))) )-

80*@if((@sum(supplier(i):kkk(i)+rkkk(i)-@sum(qcustomer(j):vkkk(i,j))) )#le#0, 0, @sum(supplier(i):kkk(i)+rkkk(i)-@sum(qcustomer(j):vkkk(i,j))) )-

70*@if((@sum(supplier(i):kkkk(i)+rkkkk(i)-@sum(qcustomer(j):vkkkk(i,j))) )#le#0, 0, @sum(supplier(i):kkkk(i)+rkkkk(i)-@sum(qcustomer(j):vkkkk(i,j))) )-

70*@if((@sum(supplier(i):kkkkk(i)+rkkkkk(i)-@sum(qcustomer(j):vkkkkk(i,j))) )#le#0, 0, @sum(supplier(i):kkkkk(i)+rkkkkk(i)-@sum(qcustomer(j):vkkkkk(i,j))) )-

@sum(supplier(i):rk(i)*80000+rkk(i)*60000+rkkk(i)*50000+rkkkk(i)*30000+rkkkkk(i)*30000)-

0.00004186*@sum(linkssq(i,j):(598.03*time(i,j)*dis(i,j)*vk(i,j)+514.03*time(i,j)*dis(i,j)*vkk(i,j)+501.64*time(i,j)*dis(i,j)*vkkk(i,j)+326.88*time(i,j)*dis(i,j)*vkkkk(i,j)+175*time(i,j)*dis(i,j)*vkkkkk(i,j)))-

0.00004186*@sum(linksfs(f,i):(598.03*ftime(f,i)*fdis(f,i)*fvk(f,i)+514.03*ftime(f,i)*fdis(f,i)*fvkk(f,i)+501.64*ftime(f,i)*fdis(f,i)*fvkkk(f,i)+326.88*ftime(f,i)*fdis(f,i)*fvkkkk(f,i)+175*ftime(f,i)*fdis(f,i)*fvkkkkk(f,i)));

!set(3);

@for(qcustomer(j):

@sum(supplier(i):vol(i,j))<=demand(j));

!set(4);

@for(linkssq(i,j):

400*time(i,j)*vk(i,j)+300*time(i,j)*vkk(i,j)+200*time(i,j)*vkkk(i,j)+120*time(i,j)*vkkkk(i,j)+120*time(i,j)*vkkkkk(i,j)>=vol(i,j));

!set(5);

@for(fcustomer(f):

@sum(supplier(i):fvol(f,i))=fdemand(f));

!set(6);

@for(linksfs(f,i):

400*ftime(f,i)*fvk(f,i)+300*ftime(f,i)*fvkk(f,i)+200*ftime(f,i)*fvkkk(f,i)+120*ftime(f,i)*fvkkkk(f,i)+120*ftime(f,i)*fvkkkkk(f,i)>=fvol(f,i));

!set(7);

@for(supplier(i):

@sum(qcustomer(j):vol(i,j))<=stocko(i)+capacity(i));

!set(8);

@for(supplier(i):

stock(i)=stocko(i)+capacity(i)+@sum(fcustomer(f):fvol(f,i))-@sum(qcustomer(j):vol(i,j)));

!set(9);

@for(supplier(i):

stock(i)<=maxst(i));

!set(10);

@for(supplier(i):

@sum(qcustomer(j):vk(i,j))<=k(i)+rk(i));

@for(supplier(i):

@sum(qcustomer(j):vkk(i,j))<=kk(i)+rkk(i));

@for(supplier(i):

@sum(qcustomer(j):vkkk(i,j))<=kkk(i)+rkkk(i));

@for(supplier(i):

@sum(qcustomer(j):vkkkk(i,j))<=kkkk(i)+rkkkk(i));

@for(supplier(i):

@sum(qcustomer(j):vkkkkk(i,j))<=kkkkk(i)+rkkkkk(i));

!set(11);

@for(supplier(i):

@sum(fcustomer(f):fvk(f,i))<=k(i)+rk(i));

@for(supplier(i):

@sum(fcustomer(f):fvkk(f,i))<=kk(i)+rkk(i));

@for(supplier(i):

@sum(fcustomer(f):fvkkk(f,i))<=kkk(i)+rkkk(i));

@for(supplier(i):

@sum(fcustomer(f):fvkkkk(f,i))<=kkkk(i)+rkkkk(i));

@for(supplier(i):

@sum(fcustomer(f):fvkkkkk(f,i))<=kkkkk(i)+rkkkkk(i));

!set(12)INT;

@for(linkssq(i,j):

@gin(vol(i,j)));

@for(linksfs(f,i):

@gin(fvol(f,i)));

@for(linkssq(i,j):

@gin(vk(i,j)));

@for(linkssq(i,j):

@gin(vkk(i,j)));

@for(linkssq(i,j):

@gin(vkkk(i,j)));

@for(linkssq(i,j):

@gin(vkkkk(i,j)));

@for(linkssq(i,j):

@gin(vkkkkk(i,j)));

@for(linksfs(f,i):

@gin(fvk(f,i)));

@for(linksfs(f,i):

@gin(fvkk(f,i)));

@for(linksfs(f,i):

@gin(fvkkk(f,i)));

@for(linksfs(f,i):

@gin(fvkkkk(f,i)));

@for(linksfs(f,i):

@gin(fvkkkkk(f,i)));

@for(supplier(i):

@gin(k(i)));

@for(supplier(i):

@gin(kk(i)));

@for(supplier(i):

@gin(kkk(i)));

@for(supplier(i):

@gin(kkkk(i)));

@for(supplier(i):

@gin(kkkkk(i)));

@for(supplier(i):

@gin(rk(i)));

@for(supplier(i):

@gin(rkk(i)));

@for(supplier(i):

@gin(rkkk(i)));

@for(supplier(i):

@gin(rkkkk(i)));

@for(supplier(i):

@gin(rkkkkk(i)));

end
