# Supplementary material for: An optimization model for fleet sizing and empty pallet allocation considering CO2 emissions
Source: PLoS One. 2020 Feb 21;15(2):e0229544. doi: 10.1371/journal.pone.0229544 (PMC7034922; doi:10.1371/journal.pone.0229544)
Supplement: S1 Table — (DOCX) [file pone.0229544.s003.docx]

**S1Table. Mathematical notations.**

| Notations | Meaning |
| --- | --- |
| $t\in T$ | Time period. |
| $i\in I$ | Pallet rental service station. |
| $j\in J$ | Demand area. Customers in area $j$ require pallets. |
| $o\in O$ | Supply area. The pallet rental service provider needs to retrieve pallets from the customers in area $o$. |
| $p\in P$ | Pallet type. |
| $k\in K$ | Vehicle type. |
| $X_{ijp}^{t}$ | The number of $p$ pallets moved from $i$ to $j$ in period $t$. |
|  | The number of $p$ pallets moved from $o$ to $i$ in period $t$. |
| $v_{ijk}^{t}$ | The number of $k$ vehicles used to transport pallets from $i$ to $j$ in period $t$. |
| $v_{oik}^{t}$ | The number of $k$ vehicles used to transport pallets from $o$ to $i$ in period $t$. |
| $v_{ik}$ | The number of $k$ vehicles configured for $i$. |
| $\theta_{ik}^{t}$ | The number of $k$ vehicles rented by $i$ in period $t$. If there are not enough vehicles to transport pallets, managers can rent vehicles from rental companies. |
| $\xi_{p}$,$C_{k}$,$\alpha_{k}$ | $\xi_{p}$ indicates the rental fee of a $p$ pallet. $C_{k}$ represents the price of purchasing a $k$ vehicle. $\alpha_{k}$ is the rental fee of a $k$ vehicle. |
| $\text{E}_{k}$,$\Lambda_{p}$,$H_{ip}$ | $\text{E}_{k}$indicates the transportation cost of a $k$ vehicle per kilometer. $\Lambda_{p}$ represents the loading and unloading cost of a $p$ pallet. $H_{ip}$ is the storage cost of a $p$ pallet at $i$. |
| $\beta_{k}$ | The idle cost of a $k$ vehicle. Pallet rental companies have to pay the maintenance fee, capital cost, and some other costs even if the purchased vehicles are not in use. |
| $l_{ij}$,$l_{oi}$ | $l_{ij}$ indicates the distance from $i$ to $j$. $l_{oi}$ indicates the distance from $o$ to $i$. |
| $B_{k}$ | The amount of carbon dioxide (CO_2_) emissions from a $k$ vehicle per kilometer. |
| $z_{k}$ | Carrying capacity. $z_{k}$ indicates how many standard pallets a $k$ vehicle can carry. |
| $g_{ijk}^{t}$，$g_{oik}^{t}$ | Turnover times. $g_{ijk}^{t}$ ($g_{oik}^{t}$) indicates how many times a $k$ vehicle can run from $i$ to $j$ (from $o$ to $i$) in period $t$. |
| $S_{ip}^{t}$ | The number of $p$ pallets purchased by $i$ in period $t$. |
| $D_{jp}^{t}$ | The number of $p$ pallets requested by $j$ in period $t$. It is certain. This kind of demand is named as deterministic demand. |
| $\Gamma_{jp}^{t}$ | The number of $p$ pallets requested by $j$ in period $t$. It is uncertain yet. This kind of demand is named as uncertain demand. |
| $S_{op}^{t}$ | The number of $p$ pallets needed to be taken back from $o$ in period $t$. It is certain. |
| $ϒ_{op}^{t}$ | The number of $p$ pallets needed to be retrieved from $o$ in period $t$. It is uncertain. |
| $M_{ip}^{t}$,$\Psi_{i}$ | $M_{ip}^{t}$ is the storage of $p$ pallets at $i$ in period $t$. $\Psi_{i}$ is the storage capacity at $i$. |
| $\upsilon_{p}$,$\psi_{p}$ | $\upsilon_{p}$ indicates the storage capacity occupied by a $p$ pallet. $\psi_{p}$ represents the carrying capacity occupied by a $p$ pallet. |
